# Diagnostic value of combining preoperative inflammatory markers ratios with CA199 for patients with early-stage pancreatic cancer

**DOI:** 10.1186/s12885-023-10653-4

**Published:** 2023-03-10

**Authors:** Yuanlong Gu, Qianjin Hua, Zhipeng Li, Xingli Zhang, Changjie Lou, Yangfen Zhang, Wei Wang, Peiyuan Cai, Juan Zhao

**Affiliations:** 1 Department of Interventional Oncology, Municipal Hospital Affiliated to Taizhou University, Taizhou, China; 2 Biotherapy Center, Harbin Medical University Cancer Hospital, Harbin, China; 3 School of Medicine, Taizhou University, Taizhou, 318000 Zhejiang China

**Keywords:** Early-stage pancreatic cancer, Diagnosis, Inflammatory, FAR, FPR, FLR

## Abstract

**Background:**

An early diagnosis of pancreatic cancer (PC) is extremely difficult because of the lack of sensitive liquid biopsy methods and effective biomarkers. We attempted to evaluate whether circulating inflammatory marker could complement CA199 for the detection of early-stage PC.

**Methods:**

We enrolled 430 patients with early-stage PC, 287 patients with other pancreatic tumors (OPT), and 401 healthy controls (HC). The patients and HC were randomly divided into a training set (*n* = 872) and two testing sets (n_1_ = 218, n_2_ = 28). The receiver operating characteristic (ROC) curves were investigated to evaluate the diagnostic performance of circulating inflammatory markers ratios, CA199, and combinations of the markers ratios in the training set, which would then be validated in the two testing sets.

**Results:**

Circulating fibrinogen, neutrophils, and monocytes in patients with PC were significantly higher while circulating albumin, prealbumin, lymphocytes, and platelets of patients with PC were significantly lower compared to those of HC and OPT (all *P* < 0.05). The fibrinogen-to-albumin (FAR), fibrinogen-to-prealbumin (FPR), neutrophil-to-lymphocyte (NLR), platelet-to-lymphocyte (PLR), monocyte-to-lymphocyte (MLR), and fibrinogen-to-lymphocyte (FLR) ratios were significantly higher while the prognostic nutrition index values (PNI) were lower in patients with PC than in HC and OPT (all *P* < 0.05). Combining the FAR, FPR, and FLR with CA199 exhibited the best diagnostic value for distinguishing patients with early-stage PC from HC with an area under the curve (AUC) of 0.964, and for distinguishing patients with early-stage PC from OPT with an AUC of 0.924 in the training sets. In the testing set, compared with HC, the combination markers had powerful efficiency for PC with an AUC 0.947 and AUC 0.942 when comparing PC with OPT. The AUC was 0.915 for the combination of CA199, FAR, FPR, and FLR for differentiating between patients with pancreatic head cancer (PHC) and other pancreatic head tumors (OPHT), and 0.894 for differentiating between patients with pancreatic body and tail cancer (PBTC) and other pancreatic body and tail tumors (OPBTT).

**Conclusion:**

A combination of FAR, FPR, FLR, and CA199 may serve as a potential non-invasive biomarker for differentiating early-stage PC from HC and OPT, especially early-stage PHC.

**Supplementary Information:**

The online version contains supplementary material available at 10.1186/s12885-023-10653-4.

## Introduction

Pancreatic cancer (PC) is the seventh leading cause of cancer-related deaths in both men and women with nearly equal rates of annual incidence and mortality [1]; it has been projected that by 2030, PC will be the second leading cause of cancer-related deaths, surpassing breast cancer, prostate, and colorectal cancers [2]. Surgical resection remains the primary form of treatment for patients with PC [3]. Currently, the diagnosis of PC is mainly based on clinical signs and symptoms, imaging techniques, serum CA199, and pathological features. However, most patients with PC are already at an advanced stage when they first visit the hospital, losing the opportunity for surgery, with a five-year survival rate of < 5% [4]. Thus, more reliable diagnostic biomarkers are urgently needed to improve early diagnosis of PC.

In recent years, liquid biopsies to isolate circulating tumor DNA (ctDNA) [5], circulating tumor cells (CTCs) [6], circulating exosomal miRNA [7], and exosomal GPC1 [8] for the early detection of PC have re ceived much attention. However, these methods are complex, time-consuming, expensive, and difficult to perform. Tumor-promoting inflammation is the seventh most important feature of cancer cells [9]. Circulating inflammatory markers such as C-reactive protein (CRP) [10], neutrophils [11], lymphocytes, platelets, monocytes [12], and fibrinogen [13] play an essential role in the oncogenesis and development of cancer. Some studies have found that inflammation markers ratios could predict the prognoses of patients with PC. For example, CRP-to-albumin score, the Glasgow Prognostic Score (GPS) each have an independent prognostic value in patients with PC [14]. A high neutrophil-to-lymphocyte ratio (NLR) is associated with an adverse overall survival (OS) in pancreatic cancer [15]. A low fibrinogen-to-albumin ratio (FAR) was positively correlated with a good OS in locally advanced or metastatic PC [16].

Notably, inflammation is evident at the earliest stages of tumor progression and could promote the development of incipient tumors into full-blown cancers [17]. Therefore, we hypothesized that these circulating inflammatory markers change within the early stages of cancer and could act as reliable indicators for early diagnoses of PC. In this study, we assessed inflammation indicator values including FAR, fibrinogen-to-prealbumin ratio (FPR), NLR, platelet-to-lymphocyte ratio (PLR), monocyte-to-lymphocyte ratio (MLR), and prognostic nutritional index (albumin + 5 × lymphocyte count; PNI) in early-stage PC, healthy controls (HC), and other pancreatic tumors (OPT), with the aim of exploring whether inflammation indicators could be used as markers for the diagnosis of early-stage PC.

## Methods

### Patients collection

This study included 422 patients with PC, 119 patients with benign pancreatic tumors (BPT; 39 chronic pancreatitis, 56 pancreatic serous cystadenomas, and 24 pancreatic mucinous cystadenomas), 98 patients with solid pseudo-papilloma of the pancreas (SPT), 59 patients with pancreatic neuroendocrine tumors (PNET), and 392 healthy controls (HC) from January 2015 to December 2021 at the Harbin Medical University Cancer Hospital. Eight patients with PC, 11 with other pancreatic diseases (OPT; two CP, two SPT, and seven pancreatic serous or mucinous cystadenoma), and nine HC from January 2017 to December 2021 in the Municipal Hospital Affiliated to Taizhou University were also enrolled in this study. The inclusion and exclusion criteria were as follows:1) age ≥ 18 years; 2) pathologically confirmed diagnoses of PC(adenocarcinoma, pancreatic ductal adenocarcinoma, and mucinous adenocarcinoma), neuroendocrine tumor (G1, G2, and G3), solid pseudopapillary neoplasm, chronic pancreatitis, pancreatic serous cystadenoma, and pancreatic mucinous cystadenoma; 3) R0 resection (radical surgical resection); 4) PC pathology at TNM stage I—II; 5) available clinical baseline information; 6) no antitumor therapy performed before surgery; 7) no second primary cancer; 8) no history of autoimmune disorders, hepatitis, nephropathy, coagulation disorders, or HIV infection; and 9) no acute inflammation before surgery.

Each disease group and HC from Harbin Medical University Cancer Hospital were randomly divided into training and testing sets 1 at a ratio of 4:1. The patients and HC from Municipal Hospital Affiliated to Taizhou University were used as testing set 2. Ethical approval for this study was granted by the Harbin Medical University Cancer Hospital and Municipal Hospital Affiliated to Taizhou University Ethics Committee, and all participants provided signed informed consent forms.

### Data collection

Detailed baseline and clinicopathological information, including sex, age, tumor location, tumor size, pathological type, differentiation, lymph node metastasis, and TNM stage of the patients with pancreatic diseases and HC, were obtained from the medical records of the inpatients or outpatients. The preoperative hematological parameters and liver function tests included neutrophils (× 10^9^/L), lymphocytes (× 10^9^/L), monocytes (× 10^9^/L), platelets (× 10^9^/L), plasma fibrinogens (g/L), serum albumins (g/L), prealbumin (mg/L), and CA199 (U/L) within seven days before surgery (average 2—7 days) were gathered from the medical records. TNM staging was performed using the 8th edition of the AJCC Cancer Staging Manual for Pancreatic Cancer.

### Inflammation markers ratios definitions

FAR, FPR, NLR, PLR, MLR, and FLR were defined as the plasma fibrinogen value divided by the serum albumin value, plasma fibrinogen value divided by the serum prealbumin value, neutrophil count divided by the lymphocyte count, platelet count divided by the lymphocyte count, monocyte count divided by the lymphocyte count, and plasma fibrinogen value divided by the lymphocyte count, respectively. PNI was defined as serum albumin value + 5 × lymphocyte count.

### Statistical analysis

Data were presented as mean ± standard deviation (SD). The differences in inflammatory markers and inflammatory markers ratios in different groups were examined using the Student’s t-test. A two-sided *p* < 0.05 was considered statistically significant. The receiver operating characteristic (ROC) curve and the area under the ROC curve (AUC) were used to evaluate the diagnostic accuracy of the inflammation indicator and CA199 for early-stage PC and the discrimination ability between early-stage PC and PNET, SPT, and BPT. ROC curve analysis was also used to determine the best cut-off values for FAR, FPR, NLR, PLR, MLR, PNI, FLR, and CA199 based on the maximum Youden index. AUC values < 0.7, 0.7—0.9, and > 0.9 were considered as low, medium, and high diagnostic power, respectively. All statistical analyses were conducted using SPSS (version23.0, IBM Corp., Armonk, NY, USA) and GraphPad Prism (version 5.0, La Jolla, CA, USA).

## Results

### Clinical characteristics and circulating inflammatory markers of patients with pancreatic diseases and HC

A total of 338 patients with early-stage PC, 96 with BPT, 78 with SPT, 47 with PNET, and 313 HC were assigned to the training set. Among the patients with PC, 187 (55.3%) were male, and the average age was 57.5 ± 8.3 years, whereas among 78 patients with SPT, 66 (84.6%) were female, and the average age was 35.5 ± 14.1 years. Most patients with SPT were young women. Most patients with PC had tumors located in the pancreatic head (76.9%), whereas most patients with BPT, SPT, and PNET had tumors located in the pancreatic body and tail cysts (76, 67.9, and 72.3%, respectively). Most patients with PC had invasive ductal carcinomas (91.1%). The clinical and pathological characteristics of the patients in the training and testing sets were similar. Detailed information on the patients and HC in the training and testing sets are listed in Table 1.

**Table 1:** Clinical characteristics of patients with pancreatic diseases and healthy controls in training and testing sets

	**Training set**				
**Groups**	**PC (338)**	**HC (313)**	**BPT (96)**	**SPT (78)**	**PNET (47)**
Gender					
Male	187(55.3)	164(52.3)	25(26.0)	12(15.4)	27(57.4)
Female	151(44.7)	149(47.6)	71(74.0)	66(84.6)	20(42.6)
Age					
≤ 60	215(63.6)	205(65.4)	70(72.9)	70(89.7)	20(42.6)
> 60	123(36.4)	103(34.5)	26(27.1)	8(10.3)	27(57.4)
CA199					
≥ 37	240(71.0)	5(1.6)	13(13.5)	4(5.1)	2(4.3)
< 37	98(29.0)	308(98.4)	83(86.5)	74(94.9)	45(95.7)
Location:					
Head	260(76.9)		23(24.0)	25(32.1)	13(27.7)
Body or Tail	78(23.1)		73(76.0)	53(67.9)	34(72.3)
Tumor size					
> 4 cm	81(24.0)		40(41.6)	40(51.3)	16(34.0)
≤ 4 cm	257(76.0)		56(58.4)	38(48.7)	31(66.0)
Pathological type					
Ductal adenocarcinoma	308(91.1)		31(32.3; chronic pancreatitis)		
others	30(8.9)		65(67.7; adenoma)		
Differentiation					
High and Moderate	218(64.5)				40(85.1; G1-G2)
Poor	120(35.5)				7(14.94; G3)
Lymph nodes					
+	107(31.7)				
-	231(68.3)				
TNM stage					
I	166(49.1)				
II	172(50.9)				
Fibrinogen(g/L)	3.44 ± 0.95	2.87 ± 0.56	2.76 ± 0.76	2.46 ± 0.69	2.54 ± 0.58
albumin(g/L)	38.52 ± 4.04	43.75 ± 2.38	39.62 ± 3.45	40.79 ± 3.72	40.69 ± 3.11
prealbumin(mg/L)	215.17 ± 69.86	322.74 ± 58.73	255.85 ± 61.72	239.98 ± 56.07	271.97 ± 65.09
neutrophil(× 10^9^/L)	3.95 ± 1.64	3.45 ± 1.05	3.41 ± 1.95	3.70 ± 1.47	3.45 ± 1.24
lymphocyte(× 10^9^/L)	1.62 ± 0.62	1.94 ± 0.56	2.03 ± 0.63	2.09 ± 0.54	1.98 ± 0.59
platelet(× 10^9^/L)	224.43 ± 69.06	238.48 ± 52.91	227.42 ± 67.02	248.58 ± 75.31	212.57 ± 51.78
monocyte(× 10^9^/L)	0.51 ± 0.19	0.37 ± 0.11	0.46 ± 0.19	0.52 ± 0.20	0.43 ± 0.16
AFR (Mean ± SD)	0.091 ± 0.03	0.066 ± 0.013	0.71 ± 0.02	0.061 ± 0.017	0.063 ± 0.01
APR (Mean ± SD)	0.019 ± 0.012	0.009 ± 0.003	0.011 ± 0.005	0.011 ± 0.006	0.009 ± 0.003
NLR (Mean ± SD)	2.78 ± 1.67	1.89 ± 0.74	1.81 ± 1.26	1.84 ± 0.77	1.87 ± 0.83
PLR (Mean ± SD)	156.32 ± 75.68	131.15 ± 45.19	119.15 ± 40.90	123.36 ± 42.09	115.21 ± 40.50
MLR (Mean ± SD)	0.36 ± 0.21	0.20 ± 0.06	0.24 ± 0.11	0.26 ± 0.10	0.23 ± 0.09
PNI (Mean ± SD)	46.62 ± 5.33	53.47 ± 3.81	49.79 ± 4.50	51.23 ± 4.92	50.59 ± 4.64
FLR (Mean ± SD)	2.46 ± 1.26	1.57 ± 0.54	1.47 ± 0.56	1.26 ± 0.51	1.38 ± 0.44

	**Testing set 1**				
**Groups**	**PC (84)**	**HC (79)**	**BPT (23)**	**SPT (20)**	**PNET (12)**
Gender					
Male	46(54.8)	46(58.2)	9(39.1)	4(20.0)	8(66.7)
Female	38(45.2)	33(41.8)	14(60.9)	16(80.0)	4(33.3)
Age					
≤ 60	44(52.4)	56(70.9)	13(56.6)	17(85.0)	7(58.3)
> 60	40(47.6)	23(29.1)	10(43.4)	3(15.0)	5(41.7)
CA199					
≥ 37	63(75.0)	2(2.6)	9(39.1)	1(5.0)	2(16.7)
< 37	21(35.0)	77(97.4)	14(60.8)	19(95.0)	10(83.3)
Location:					
Head	58(69.0)		4(17.4)	5(25.0)	5(41.7)
Body or Tail	34(31.0)		19(82.6)	15(75.0)	7(58.3)
Tumor size					
> 4 cm	21(44.7)		11(47.8)	11(55.0)	4(33.3)
≤ 4 cm	63(55.3)		12(52.2)	9(45.0)	8(66.7)
Pathological type					
Ductal adenocarcinoma	75(89.3)		8(34.7; chronic pancreatitis)		
others	9(10.7)		15(65.2; adenoma)		
Differentiation					
High and Moderate	43(51.2)				10(83.3; G1-G2)
Poor	41(48.8)				2(16.7; G3)
Lymph nodes					
+	17(20.2)				
-	67(79.8)				
TNM stage					
I	50(59.5)				
II	34(40.5)				
Fibrinogen(g/L)	3.56 ± 0.94	2.82 ± 0.49	2.59 ± 0.58	2.42 ± 0.60	2.59 ± 0.69
albumin(g/L)	37.98 ± 3.07	43.78 ± 2.09	38.68 ± 3.23	40.54 ± 3.25	40.5 ± 3.28
prealbumin(mg/L)	212.73 ± 45.03	312.30 ± 57.40	269.65 ± 63.85	231.74 ± 55.87	280.17 ± 52.37
neutrophil(× 10^9^/L)	3.88 ± 1.26	3.46 ± 0.95	3.37 ± 1.24	3.24 ± 1.12	3.17 ± 1.15
lymphocyte(× 10^9^/L)	1.59 ± 0.45	2.01 ± 0.66	1.89 ± 0.44	2.05 ± 0.76	1.84 ± 0.42
platelet(× 10^9^/L)	234.04 ± 77.48	250.29 ± 61.98	234.26 ± 78.76	241.37 ± 49.04	198.08 ± 46.20
monocyte(× 10^9^/L)	0.49 ± 0.14	0.37 ± 0.10	0.42 ± 0.14	0.42 ± 0.13	0.40 ± 0.14
AFR (Mean ± SD)	0.095 ± 0.028	0.065 ± 0.01	0.067 ± 0.015	0.060 ± 0.015	0.064 ± 0.015
APR (Mean ± SD)	0.018 ± 0.009	0.009 ± 0.003	0.010 ± 0.003	0.011 ± 0.005	0.009 ± 0.002
NLR (Mean ± SD)	2.57 ± 0.85	1.85 ± 0.65	1.83 ± 0.72	1.73 ± 0.75	1.74 ± 0.59
PLR (Mean ± SD)	157.09 ± 60.35	133.85 ± 45.32	128.29 ± 49.03	135.00 ± 57.96	112.36 ± 34.29
MLR (Mean ± SD)	0.33 ± 0.13	0.20 ± 0.06	0.22 ± 0.06	0.24 ± 0.15	0.22 ± 0.07
PNI (Mean ± SD)	45.91 ± 3.88	53.83 ± 3.95	48.16 ± 4.11	50.78 ± 5.13	49.70 ± 4.67
FLR (Mean ± SD)	2.45 ± 0.97	1.55 ± 0.55	1.45 ± 0.50	1.34 ± 0.59	1.46 ± 0.47

	**Testing set 2**		
**Groups**	**PC (8)**	**HC (9)**	**BPT (11)**
Gender			
Male	5(62.5)	7(77.8)	6(54.5)
Female	3(37.5)	2(22.2)	5(45.4)
Age			
≤ 60	5(62.5)	6(66.7)	6(54.5)
> 60	3(37.5)	3(33.3)	5(45.4)
CA199			
≥ 37	6(75.0)	1(11.1)	1(9.1)
< 37	2(25.0)	8(88.9)	10(90.9)
Location:			
Head	6(75.0)		4(36.4)
Body or Tail	2(25.0)		7(63.6)
Tumor size			
> 4 cm	2(25.0)		5(45.5)
≤ 4 cm	6(75.0)		6(54.4)
Pathological type			
Ductal adenocarcinoma	7(87.5)		2(18.2; chronic pancreatitis) 7 (63.6; adenoma) 2(18.2, Solid pseudo papilloma)
others	1(12.5)		
Differentiation			
High and Moderate	5(62.5)		
Poor	3(37.5)		
Lymph nodes			
+	3(37.5)		
-	5(62.5)		
TNM stage			
I	4(50.0)		
II	4(50.0)		
Fibrinogen(g/L)	3.16 ± 0.55	2.55 ± 0.54	2.81 ± 0.52
albumin(g/L)	38.20 ± 3.08	40.83 ± 4.49	43.66 ± 3.62
prealbumin(mg/L)	205.25 ± 22.32	287.78 ± 15.70	278.72 ± 10.51
neutrophil(× 10^9^/L)	3.69 ± 0.86	2.97 ± 0.71	2.75 ± 0.71
lymphocyte(× 10^9^/L)	1.46 ± 0.34	1.95 ± 0.43	1.86 ± 0.54
platelet(× 10^9^/L)	192.5 ± 54.5	230.67 ± 48.95	237.24 ± 97.47
monocyte(× 10^9^/L)	0.48 ± 0.16	0.40 ± 0.16	0.36 ± 0.13
AFR (Mean ± SD)	0.082 ± 0.11	0.063 ± 0.015	0.65 ± 0.13
APR (Mean ± SD)	0.015 ± 0.002	0.009 ± 0.002	0.010 ± 0.002
NLR (Mean ± SD)	2.60 ± 0.73	1.58 ± 0.43	1.54 ± 0.44
PLR (Mean ± SD)	135.35 ± 40.17	121.40 ± 28.15	133.67 ± 54.68
MLR (Mean ± SD)	0.33 ± 0.81	0.20 ± 0.06	0.20 ± 0.07
PNI (Mean ± SD)	45.51 ± 3.58	50.57 ± 4.59	52.90 ± 4.44
FLR (Mean ± SD)	2.24 ± 0.56	1.35 ± 0.34	1.56 ± 0.32

*PC* pancreatic cancer, *HC* Healthy controls, *BPT* Benign pancreas tumors, *SPT* Solid pseudo papilloma of the pancreas, *PNET* Pancreatic neuroendocrine tumors, *FAR* Fibrinogen-to-albumin ratio, *FPR* Fibrinogen-to-prealbumin ratio, *NLR* Neutrophil-to-lymphocyte ratio, *PLR* Platelets-to-lymphocyte ratio, *MLR* Monocytes-to-lymphocyte ratio, *PNI* Albumin + 5 × the lymphocyte count, *FLR* Fibrinogen-to- lymphocyte ratio

**Fig. 1:**
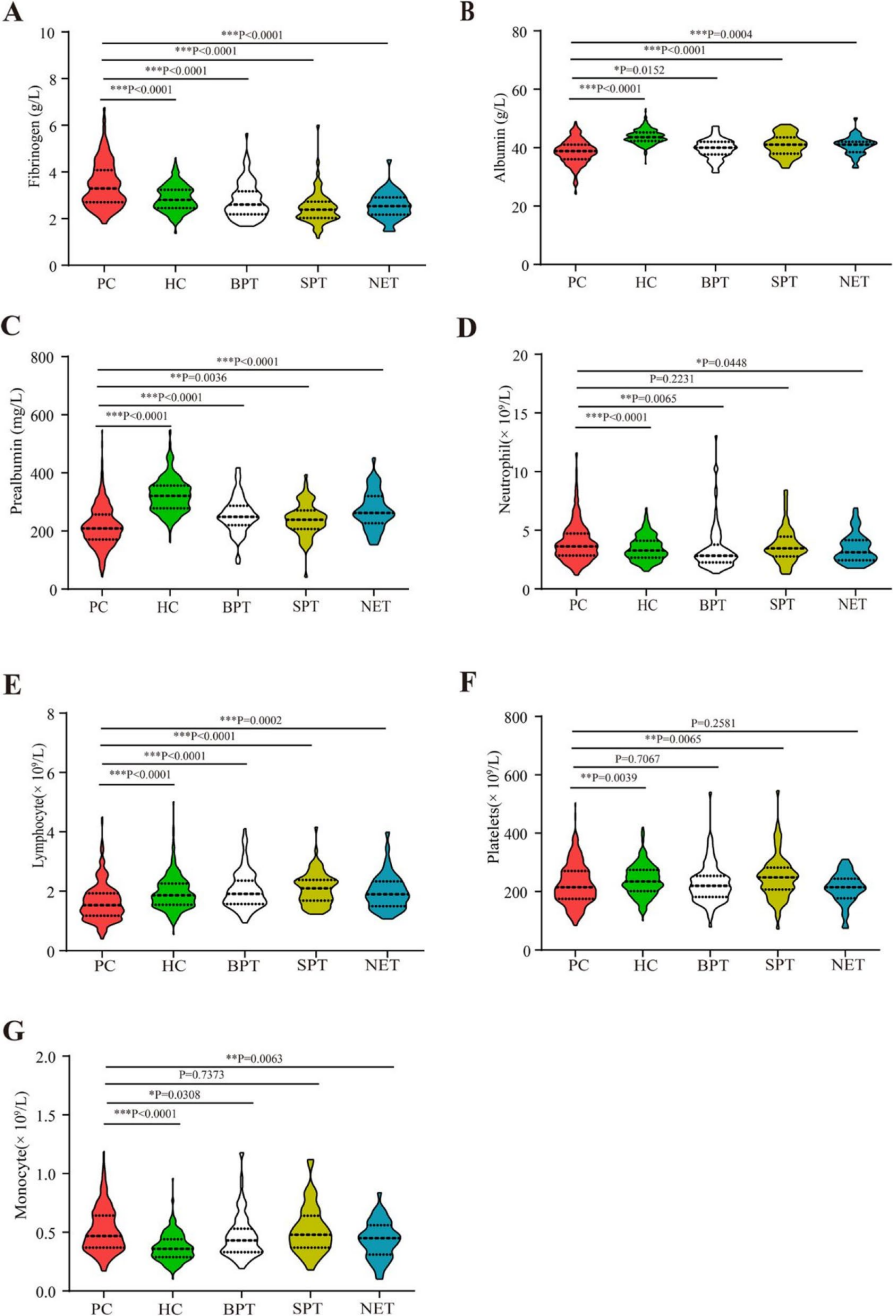
The circulating inflammation markers in PC, HC, BPT, SPT, and PNET in training sets. The plasma fibrinogens (**A**), serum albumins (**B**), prealbumin (**C**), neutrophils (**D**), lymphocytes (**E**), platelets (**F**), and monocytes (**G**) in PC, HC, BPT, SPT, and PNET. Abbreviations: PC, pancreatic cancer; BPT, benign pancreas tumors; SPT, solid pseudo papilloma of the pancreas; PNET, patients with pancreatic neuroendocrine tumors; HC, healthy controls

We compared the hematological and biochemical parameters of patients with PC, BPT, SPT, PNET, and HC. As shown in Fig. 1, in the training set, the average fibrinogen, neutrophil, platelet, and monocyte levels in patients with PC were 3.44 ± 0.95 g/L, 3.95 ± 1.64 × 10^9^/L, 224.43 ± 69.06 × 10^9^/L, and 0.51 ± 0.19 × 10^9^/L, respectively; these were significantly higher compared to those of the HC and OPT groups, with *P* values < 0.05. In contrast, the average albumin, prealbumin, lymphocytes, and platelets of patients with PC were 38.52 ± 4.04 g/L, 215.17 ± 69.86 mg/L, 1.62 ± 0.62 × 10^9^/L, 224.43 ± 69.06 × 10^9^/L, respectively, which were significantly lower than those in the HC and OPT groups, with *P* values < 0.05. The results obtained from the testing set were consistent with those obtained from the training set (Supplementary Fig. 1). These results suggest that circulating inflammatory markers had already changed in the early stages of PC.

### Inflammation markers ratios values in pancreatic diseases and HC

**Fig. 2:**
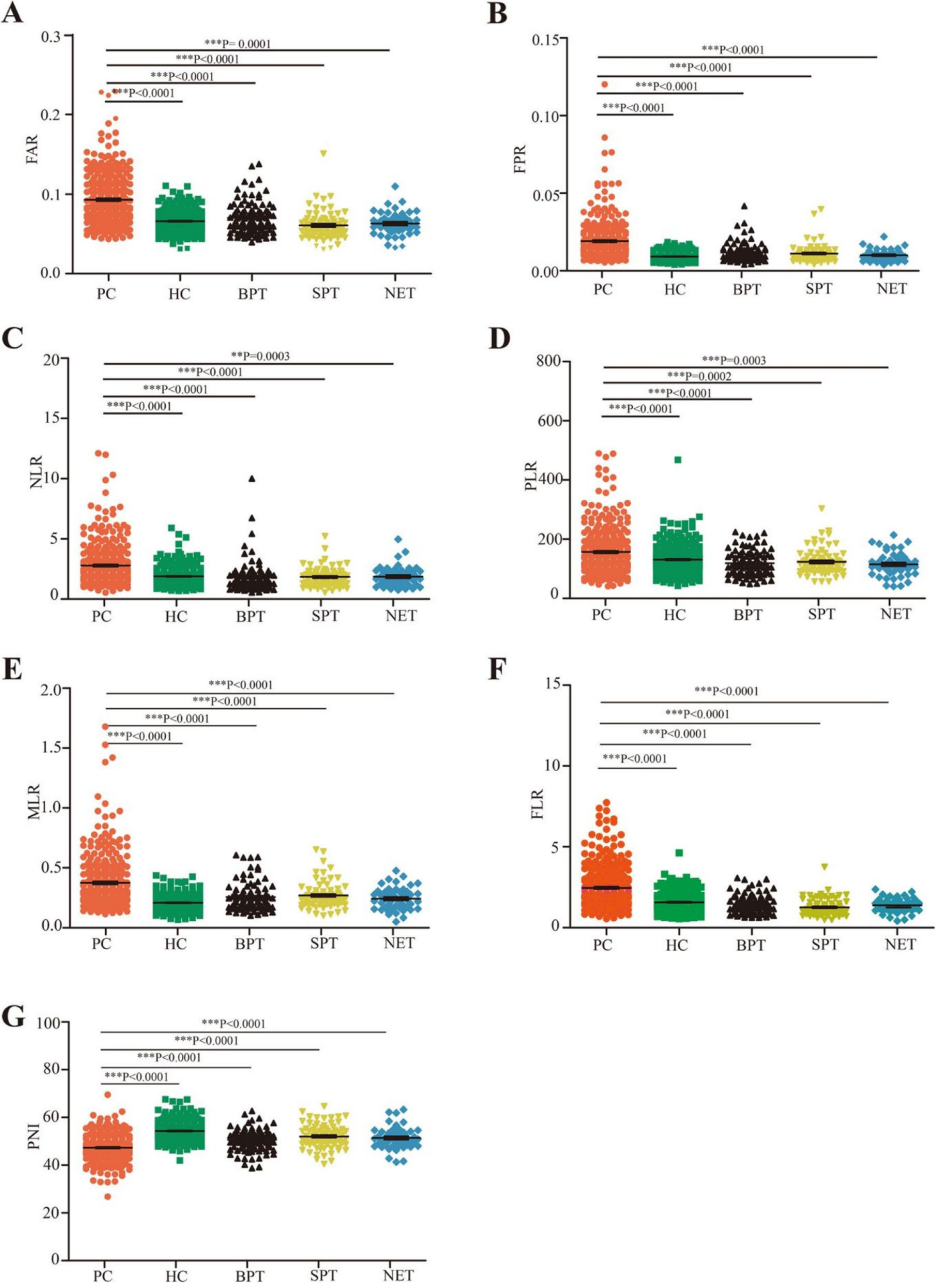
The inflammation markers ratios in PC, HC, BPT, SPT, and PNET in training sets. The FAR (**A**), FPR (**B**), NLR (**C**), PLR (**D**), MLR (**E**), FLR (**F**), and PNI (**G**) in PC, HC, BPT, SPT, and PNET. Abbreviations: PC, pancreatic cancer; BPT, benign pancreas tumors; SPT, solid pseudo papilloma of the pancreas; PNET, patients with pancreatic neuroendocrine tumors; HC, healthy controls; FAR, fibrinogen-to-albumin ratio; FPR, fibrinogen-to-prealbumin ratio; NLR, neutrophil-to-lymphocyte ratio; PLR, platelets-to-lymphocyte ratio; MLR monocytes-to-lymphocyte ratio; PNI, albumin + 5 × the lymphocyte count, FLR, fibrinogen-to- lymphocyte ratio

As shown in Table 1, in the training set, FAR, FPR, NLR, PLR, MLR, PNI, and FLR values of patients with PC were 0.091 ± 0.03, 0.019 ± 0.012, 2.78 ± 1.67, 156.32 ± 75.68, 0.36 ± 0.21, 46.62 ± 5.33, and 2.46 ± 1.26, respectively. FAR values were significantly higher in patients with PC than those in the HC, BPT, SPT, and PNET groups (Fig. 2A, *P* < 0.0001, *P* < 0.0001, *P* < 0.0001, and *P* < 0.0001, respectively). FPR values were significantly higher in patients with PC than those of HC, BPT, SPT, and PNET (Fig. 2B, *P* < 0.0001, *P* < 0.0001, *P* < 0.0001, and *P* < 0.0001, respectively). NLR values were significantly higher in patients with PC than those in the HC, BPT, SPT, and PNET groups (Fig. 2C, *P* < 0.0001, *P* < 0.0001, *P* < 0.0001, and *P* = 0.0003, respectively). PLR values were higher in patients with PC than those in the HC, BPT, SPT, and PNET groups (Fig. 2D, *P* < 0.0001, *P* < 0.0001, *P* = 0.0002, and *P* = 0.0003, respectively). MLR values were higher in patients with PC than those in the HC, BPT, SPT, and PNET groups (Fig. 2E, *P* < 0.0001, *P* < 0.0001, *P* < 0.0001, and *P* < 0.0001, respectively). FLR values were higher in patients with PC than those in the HC, BPT, SPT, and PNET groups (Fig. 2F, *P* < 0.0001, *P* < 0.0001, *P* < 0.0001, and *P* = 0.0008, respectively). In contrast, PNI values were lower in patients with PC than those in the HC, BPT, SPT, and PNET groups (Fig. 2G, *P* < 0.0001, *P* < 0.0001, *P* < 0.0001, and *P* < 0.0001, respectively). The results from the testing sets were consistent with those from the training set; the detailed data in the testing sets are shown in supplementary Fig. 2A—G. These results indicated that the inflammation markers ratios were significantly altered in patients with early-stage PC.

### Diagnostic and differential diagnosis values of inflammation markers ratios values in PC

**Fig. 3:**
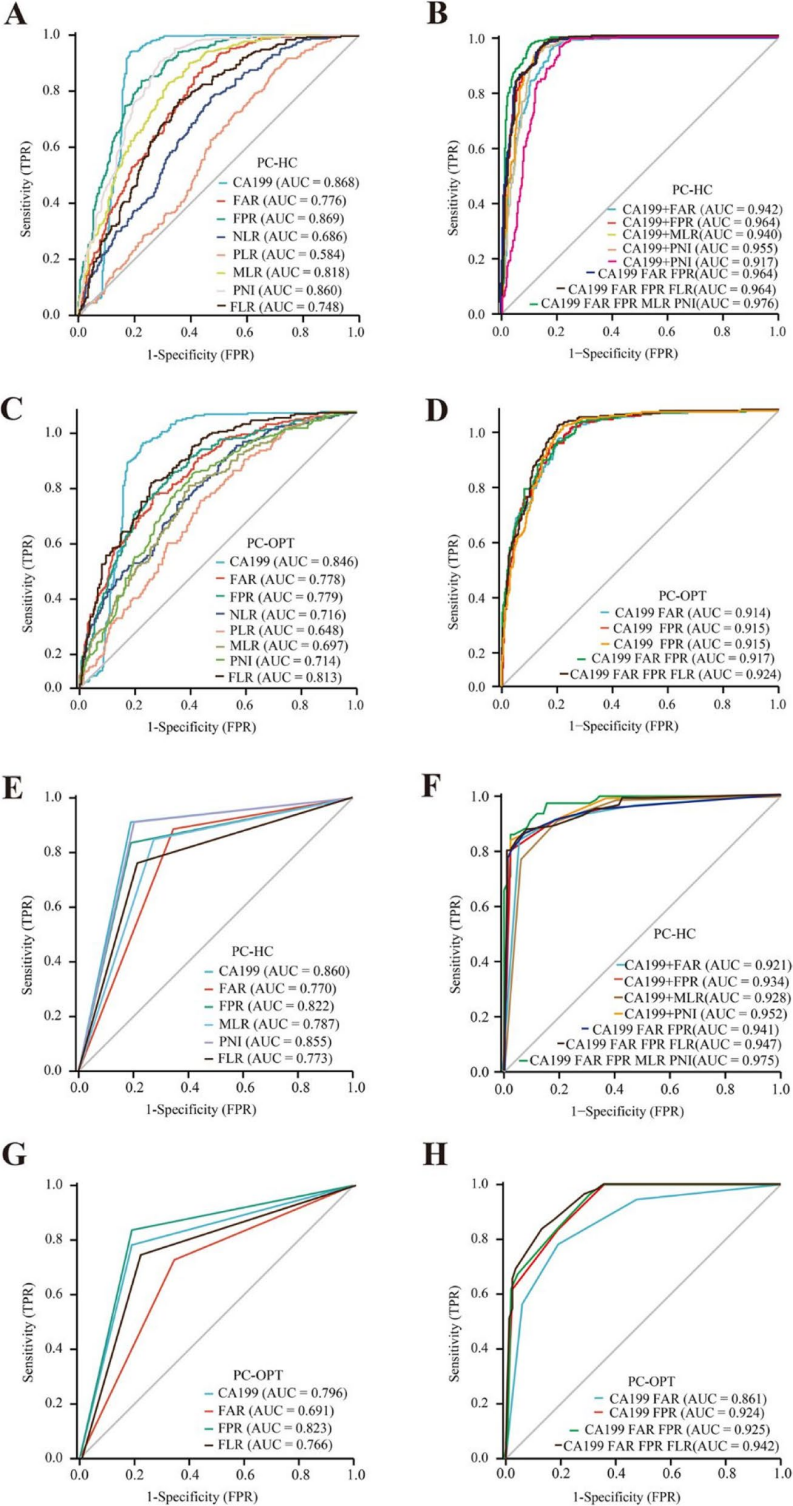
Diagnostic value of single and combined inflammation markers ratios in early-stage PC. **A** The ROC curve analysis of FAR, FPR, NLR, PLR, MLR, FLR, PNI, and CA199 between PC and HC in the training set. **B** The ROC curve analysis of combined inflammation markers ratios and CA199 in PC and HC in the training set.** C** The ROC curve analysis of FAR, FPR, NLR, PLR, MLR, FLR, PNI, and CA199 between PC and OPT in the training set. **D** The ROC curve analysis of combined inflammation indicator and CA199 between PC and OPT in the training set. **E** The ROC curve analysis of FAR, FPR, MLR, FLR, PNI, and CA199 between PC and HC in testing set 1. **F** The ROC curve analysis of combined inflammation markers ratios and CA199 between PC and HC in testing set 1. **G** The ROC curve analysis of FAR, FPR, FLR, and CA199 between PC and OPT in testing set 1. **H** The ROC curve analysis of combined inflammation markers ratios and CA199 between PC and OPT in testing set 1. Abbreviations: PC, pancreatic cancer; OPT, other pancreas tumors; HC, healthy controls; FAR, fibrinogen-to-albumin ratio; FPR, fibrinogen-to-prealbumin ratio; NLR, neutrophil-to-lymphocyte ratio; PLR, platelets-to-lymphocyte ratio; MLR monocytes-to-lymphocyte ratio; PNI, albumin + 5 × the lymphocyte count; FLR, fibrinogen-to- lymphocyte ratio; ROC, receiver operating characteristic

In the training sets, the ROC curve was used to evaluate the capabilities of CA199, FAR, FPR, NLR, PLR, MLR, and PNI in discriminating between early stage PC and HC. The AUC values were 0.868 for CA199 (*P* < 0.0001, cutoff 24.540, sensitivity 0.939, specificity 0.817), 0.776 for FAR (*P* < 0.0001, cutoff 0.080, sensitivity 0.885, specificity 0.556), 0.869 for FPR (*P* < 0.0001, cutoff 0.012, sensitivity 0.837, specificity 0.775), 0.686 for NLR (*P* < 0.0001, cutoff 2.252, sensitivity 0.780, specificity 0.527), 0.584 for PLR (*P* = 0.0002, cutoff 177.218, sensitivity 0.879, specificity 0.299), 0.818 for MLR (*P* < 0.0001, cutoff 0.249, sensitivity 0.830, specificity 0.678), 0.748 for FLR (*P* < 0.0001, cutoff 1.864, sensitivity 0.773, specificity 0.639), and 0.860 for PNI (*P* < 0.0001, cutoff 49.025, sensitivity 0.907, specificity 0.707) (Fig. 3A, Table 2). The AUC was 0.942 for a combination of CA199 and FAR, 0.964 for CA199 and FPR, 0.940 for CA199 + MLR, 0.955 for CA199 + PNI, 0.964 for CA199 + FAR + FPR, 0.964 for CA199 + FAR + FPR + FLR, and 0.976 for CA199 + FAR + FPR + MLR + PNI (Fig. 3B, Table 2). To determine whether inflammation indicator values could differentiate PC from other pancreatic diseases (OPT), we generated ROC curves. As shown in Fig. 3C-D and Table 3, the AUC was 0.846 for CA199 (*P* < 0.0001, cut-off 32.205, sensitivity 0.887, specificity 0.772), 0.778 for FAR (*P* < 0.0001, cut-off 0.070,sensitivity 0.701, specificity 0.734), 0.779 for FPR (*P* < 0.0001, cut-off 0.013, sensitivity 0.778, specificity 0.666), 0.716 for NLR (*P* < 0.0001, cut-off 1.961, sensitivity 0.674, specificity 0.642), 0.648 for PLR (*P* < 0.0001, cut-off 128.575, sensitivity 0.679, specificity 0.565), 0.697 for MLR (*P* < 0.0001, cut-off 0.271, sensitivity 0.733, specificity 0.607), 0.714 for PNI (*P* < 0.0001, cut-off 47.225, sensitivity 0.774, specificity 0.595), and 0.813 for FLR (*P* < 0.0001, cut-off 1.631, sensitivity 0.747, specificity 0.743). The AUC was 0.914 for a combination of CA199 + FAR, 0.915 for CA199 + FPR, 0.917 for CA199 + FAR + FPR, and 0.924 for CA199 + FAR + FPR + FLR. We calculated the ROC curves and AUC for the testing set 1 and testing set 2 using the best cut-off value from the ROC curve in the training set. In testing set 1, the AUC was 0.941 for a combination of CA199 + FAR + FPR, 0.947 for CA199 + FAR + FPR + FLR, 0.975 for CA199 + FAR + FPR + MLR + PNI to distinguish patients with PC from HC; 0.925 for CA199 + FAR + FPR, and 0.942 for CA199 + FAR + FPR + FLR to differentiate patients with PC from those with OPT. The results revealed that combinations of CA199 and inflammation indicator values had a strong capability for differentiating patients with PC from the HC and OPT groups, especially the combination of CA199 + FAR + FPR + FLR (Fig. 3E-H, and supplementary Tables 1 and 2). In testing set 2, the AUC was 0.993 for combination of CA199 + FAR + FPR + FLR to distinguish patients with PC from HC, and 0.994 for combination of CA199 + FAR + FPR + FLR to differentiate patients with PC from those with OPT (supplementary Fig. 3).

**Table 2:** ROC curve results based on FAR, FPR, NLR, PLR, LMR, PNI, FLR, and CA199 for distinguishing PC patients from HC in training set

Marker	AUC (95%CI)	*P*—value	**c**ut-off	Sensitivity	Specificity
FAR	0.776(0.740–0.811)	< 0.0001	0.080	0.885	0.556
FPR	0.869(0.842–0.896)	< 0.0001	0.012	0.837	0.775
NLR	0.686(0.645–0.726)	< 0.0001	2.252	0.780	0.527
PLR	0.584(0.540–0.628)	0.0002	177.218	0.879	0.299
MLR	0.818(0.786–0.850)	< 0.0001	0.249	0.831	0.678
PNI	0.860(0.831–0.888)	< 0.0001	49.025	0.907	0.707
FLR	0.748(0.711–0.785)	< 0.0001	1.864	0.773	0.639
CA199	0.868(0.836–0.901)	< 0.0001	24.540	0.939	0.817
CA199 + FAR	0.942(0.924–0.960)	< 0.0001	-0.459	0.971	0.814
CA199 + FPR	0.964(0.951–0.977)	< 0.0001	-0.470	0.978	0.840
CA199 + MLR	0.940(0.921–0.960)	< 0.0001	0.371	0.946	0.870
CA199 + PNI	0.955(0.939–0.971)	< 0.0001	0.002	0.949	0.870
CA199 + FLR	0.917(0.893–0.940)	< 0.0001	-0.035	0.965	0.793
CA199 + FAR + FPR	0.964(0.951–0.978)	< 0.0001	-0.278	0.965	0.858
CA199 + FAR + FPR + FLR	0.964(0.951–0.978)	< 0.0001	-0.466	0.974	0.849
CA199 + FAR + FPR + MLR + PNI	0.976(0.965–0.988)	< 0.0001	-0.294	0.974	0.891

*Abbreviations*: *PC* Pancreatic cancer, *HC* Heathy controls, *ROC* Receiver operating characteristic, *AUC* Area under the receiver operating characteristic curve, *CI* Confidence interval, *FPR* Fibrinogen-to-prealbumin ratio, *FAR* Fibrinogen-to-albumin ratio, *NLR* Neutrophil-to-lymphocyte ratio, *PLR* Platelets-to-lymphocyte ratio, *MLR* Monocytes-to-lymphocyte ratio, *PNI* Albumin + 5 × the lymphocyte count, *FLR* Fibrinogen-to-lymphocyte ratio

**Table 3:** ROC curve results based on FAR, FPR, NLR, PLR, LMR, PNI, FLR, and CA199 for distinguish PC patients from OPT in testing set 1

Marker	AUC (95%CI)	*P*—value	cut-off	Sensitivity	Specificity
FAR	0.778(0.740–0.817)	< 0.0001	0.070	0.701	0.734
FPR	0.779(0.740–0.817)	< 0.0001	0.013	0.778	0.666
NLR	0.716(0.674–0.759)	< 0.0001	1.961	0.674	0.642
PLR	0.648(0.603–0.694)	< 0.0001	128.575	0.679	0.565
MLR	0.697(0.654–0.741)	< 0.0001	0.271	0.733	0.607
PNI	0.714(0.671–0.757)	< 0.0001	47.225	0.774	0.595
FLR	0.813(0.778–0.848)	< 0.0001	1.631	0.747	0.743
CA199	0.846(0.812–0.880)	< 0.0001	32.205	0.887	0.772
CA199 + FAR	0.914(0.891–0.937)	< 0.0001	-0.182	0.896	0.799
CA199 + FPR	0.915(0.891–0.938)	< 0.0001	0.101	0.860	0.831
CA199 + FLR	0.915(0.829–0.938)	< 0.0001	-0.523	0.946	0.778
CA199 + FAR + FPR	0.917(0.895–0.940)	< 0.0001	-0.125	0.887	0.799
CA199 + FAR + FPR + FLR	0.924(0.903–0.946)	< 0.0001	-0.506	0.941	0.799

*Abbreviations*: *PC* Pancreatic cancer, *OPT* Other pancreas tumors, *ROC* Receiver operating characteristic, *AUC* Area under the receiver operating characteristic curve, *CI* Confidence interval, *FPR* Fibrinogen-to-prealbumin ratio, *FAR* Fibrinogen-to-albumin ratio, *NLR* Neutrophil-to-lymphocyte ratio, *PLR* Platelets-to-lymphocyte ratio, *MLR* Monocytes-to-lymphocyte ratio, *PNI* Albumin + 5 × the lymphocyte count, *FLR* Fibrinogen-to- lymphocyte ratio

### Relationship between inflammation markers ratios values and clinical characteristics of patients with PC

**Fig. 4:**
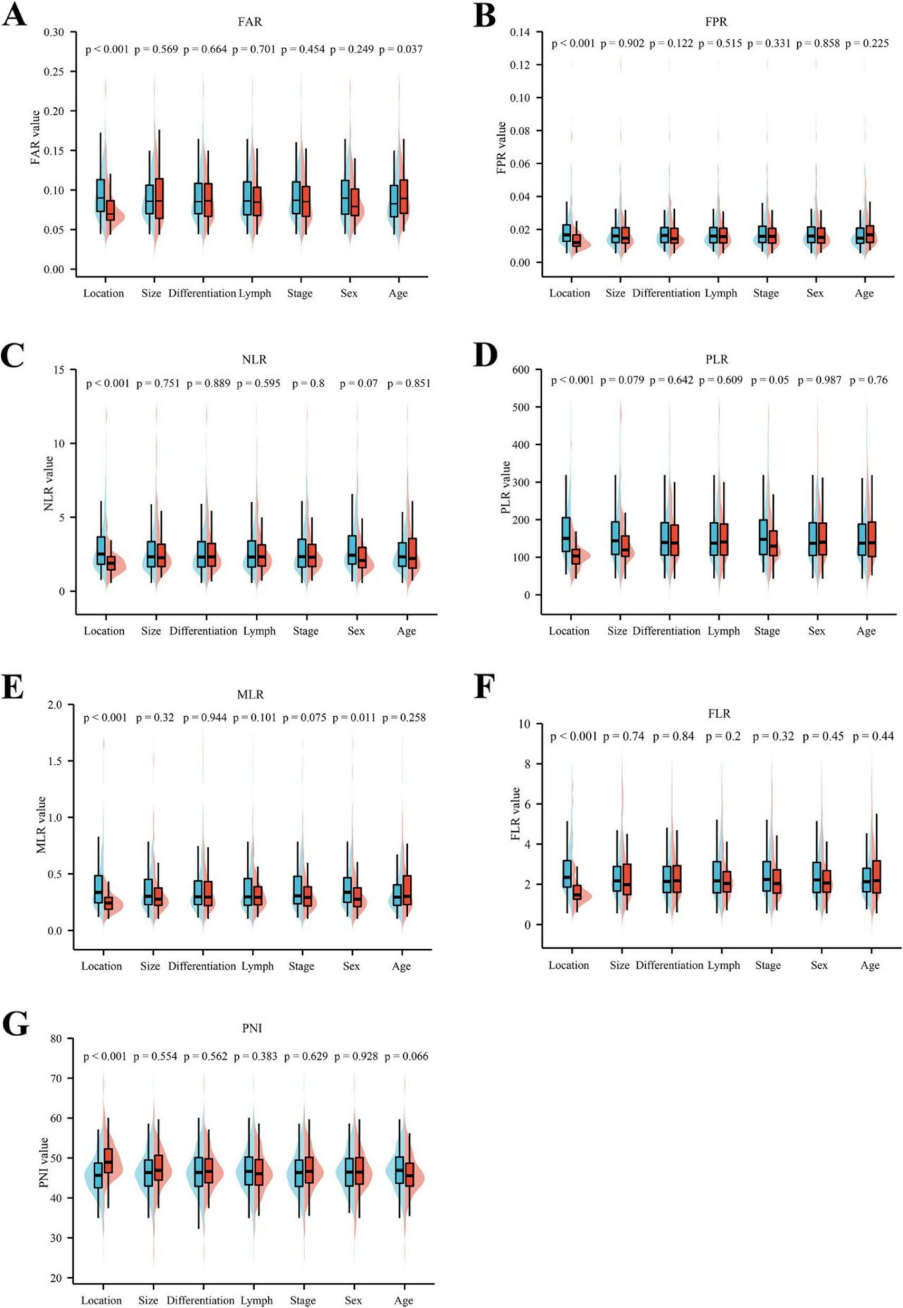
Comparison of inflammation markers ratios in different clinical characteristics early-stage PC. The FAR(**A**), FPR (**B**), NLR (**C**), PLR (**D**), MLR (**E**), FLR (**F**), and PNI (**G**), and in different tumor location, tumor size, differentiation, lymph nodes, stage, sex, and age in the training set. Abbreviations: PC, pancreatic cancer; FAR, fibrinogen-to-albumin ratio; FPR, fibrinogen-to-prealbumin ratio; NLR, neutrophil-to-lymphocyte ratio; PLR, platelets-to-lymphocyte ratio; MLR monocytes-to-lymphocyte ratio; PNI, albumin + 5 × the lymphocyte count; FLR, fibrinogen-to- lymphocyte ratio; Blue column: location, pancreatic head/tumor size ≤ 4 cm/ differentiation well / lymph node metastasis no /stage I/sex male/age ≤ 60; Red column: location, pancreatic body and tail/tumor size > 4 cm/ differentiation poor / lymph node metastasis yes /stage II/sex male/age > 60

The relationship between inflammation markers ratios and the clinical characteristics of patients with PC was analyzed. In the training set, patients with pancreatic head cancer had higher FAR, FPR, NLR, PLR, MLR, FLR, and lower PNI values than patients with pancreatic body or tail cancers (Fig. 4A-G; *P* < 0.001, *P* < 0.001, *P* < 0.001, *P* < 0.001, *P* < 0.001, and *P* < 0.001, respectively). Patients aged > 60 years had higher FAR values than those aged ≤ 60 years (Fig. 4A; *P* = 0.037). Male patients with PC had higher MLR values than female patients with PC (Fig. 4E, *P* = 0.011). In the testing set, the same trend was observed for the FAR, FPR, NLR, FLR, and PNI values (Supplementary Fig. 4A-C, F-G; *P* < 0.001, *P* = 0.007, *P* = 0.04, *P* < 0.05, and *P* = 0.004, respectively). Similarly, patients who were > 60 years of age had higher FAR, FPR, MLR, FLR, and lower PNI values than those aged ≤ 60 years (Supplementary Fig. 4A-B, E-F; *P* = 0.017, *P* = 0.007, *P* = 0.013, *P* < 0.05, and *P* = 0.02, respectively). In the two groups, the inflammation markers ratios values were independent of tumor size, differentiation, lymph nodes, TNM stage and sex (*P* > 0.05 in all inflammation markers ratios values).

### Differential diagnosis power of inflammation markers ratios values in different tumors location of PC

**Fig. 5:**
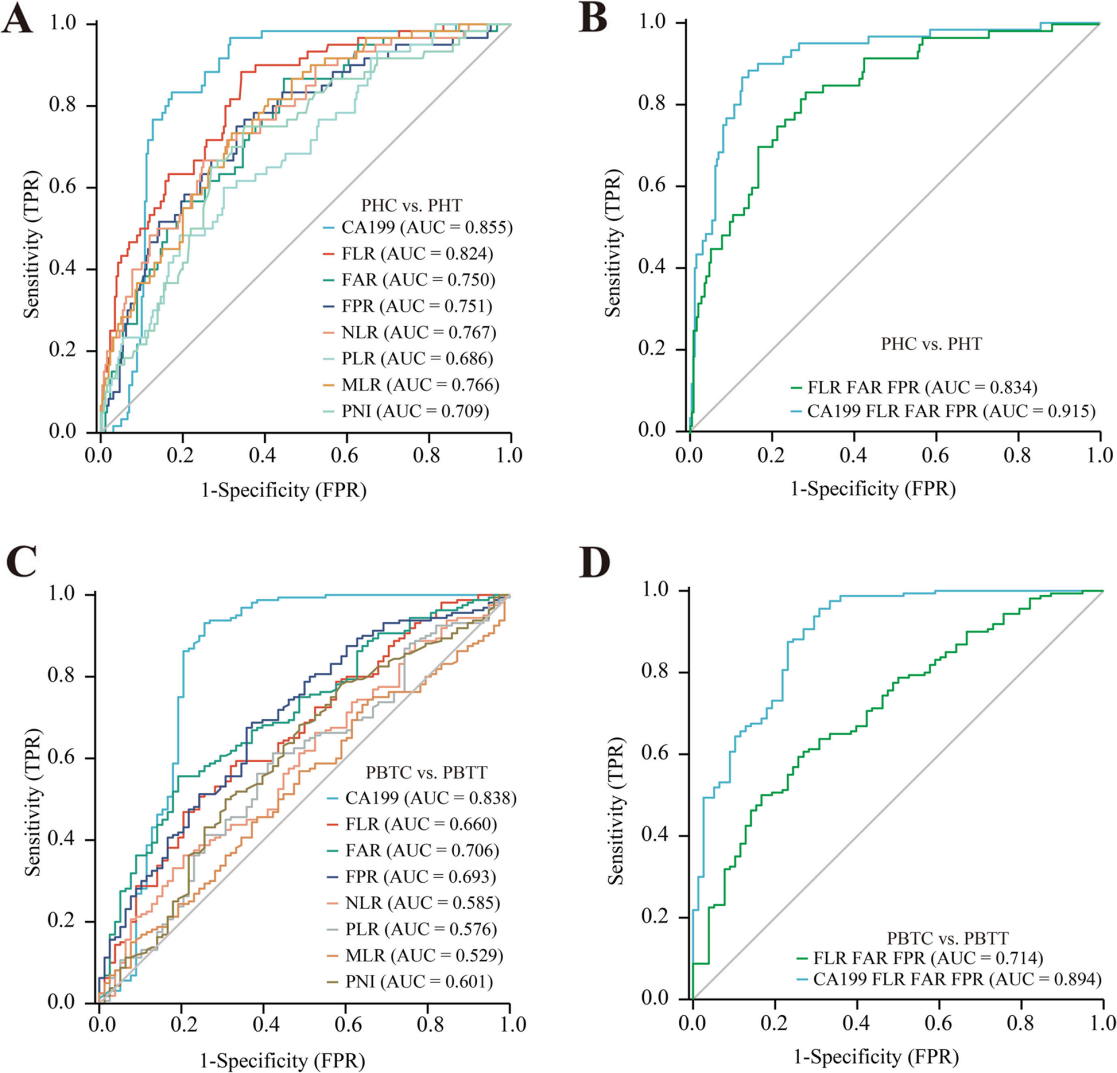
Diagnostic value of single and combined inflammation markers ratios in different tumor location PC. **A** The ROC curve analysis of FAR, FPR, NLR, PLR, MLR, PNI, FLR, and CA199 between PHC and OPHT in the training sets. **B** The ROC curve analysis of combined inflammation markers ratios and CA199 between PHC and OPHT in the training sets. **C** The ROC curve analysis of FAR, FPR, NLR, PLR, MLR, PNI, FLR, and CA199 between PBTC and OPBTT in the training sets. **D** The ROC curve analysis of combined inflammation markers ratios and CA199 between PBTC and OPBTT in the training sets. Abbreviations: PHC, pancreatic head cancer; OPHT, other pancreas head tumors; PBTC, pancreatic body, and tail cancer; OPBTT, other pancreas body, and tail tumors; FAR, fibrinogen-to-albumin ratio; FPR, fibrinogen-to-prealbumin ratio; NLR, neutrophil-to-lymphocyte ratio; PLR, platelets-to-lymphocyte ratio; MLR monocytes-to-lymphocyte ratio; PNI, albumin + 5 × the lymphocyte count; FLR, fibrinogen-to- lymphocyte ratio; ROC, receiver operating characteristic

Patients with PC and OPT were classified into four subgroups according to the locations of the pancreatic lesions: pancreatic head cancer (PHC), pancreatic body and tail cancer (PBTC), other pancreatic head tumors (OPHT), and other pancreatic body and tail tumors (OPBTT). The AUC was 0.855 for CA199, 0.750 for FAR, 0.751 for FPR, 0.824 for FLR, 0.767 for NLR, 0.686 for PLR, 0.766 for MLR, and 0.709 for PNI, to differentiate between patients with PHC and OPHT (Fig. 5A). The AUC was 0.834 for FAR + FPR + FLR and 0.915 for CA199 + FAR + FPR + FLR, to differentiate between patients with PHC and those with OPHT (Fig. 5B). The AUC was 0.838 for CA199, 0.706 for FAR, 0.693 for FPR, 0.660 for FLR, 0.585 for NLR, 0.576 for PLR, 0.529 for MLR, and 0.601 for PNI, to differentiate between patients with PHC and OPHT (Fig. 5C). The AUC was 0.714 for FAR + FPR + FLR and 0.894 for CA199 + FAR + FPR + FLR, to differentiate between patients with PBTC and OPBTT (Fig. 5D). These results showed that a combination of CA199 + FAR + FPR + FLR could better help identify PHC and OPHT.

## Discussion

Cancer-associated inflammation has been reported as the seventh hallmark of cancer [9]. Nearly all human cancers harbor inflammatory reactions, which play an important role in tumor development, progression, and metastasis [18]. Systemic inflammation can play a supporting role in the evolution of PC. For example, chronic pancreatitis is a known risk factor for the development of PC [19]. Obesity, another risk factor for pancreatic cancer, can induce inflammation by promoting the release of IL-6, CCL2, and CCL5, and the infiltration of macrophages and immunosuppressive cells [20]. Smoking is an established risk factor for PC and can induce inflammation and immune activation [21]. In addition, cancer cells can promote systemic inflammation that can, in turn, support tumor growth and lead to a poor prognosis in PC [18]. Inflammatory cells and chemokines shape the inflammatory microenvironment leading to cancer [22]. For example, IL-6, IL-1β, and TNF-α were increased at cancer early stage and associated with disease severity [23]. In Pancreatic ductal adenocarcinoma (PDAC) mouse models, adipocyte-secreted IL-1β could promote obesity-induced pancreatic carcinogenesis and drug resistance through recruitment of tumor-associated neutrophils [24]. High intra-tumoral and serum IL-1β levels in PC patients correlate with poor overall survival and increased chemoresistance [25]. IL-6, a pro-inflammatory cytokine that shows diverse functions of cell multiplication, injury, infection, and inflammation, affects tumor cells to develop PC by controlling vascular endothelial growth factor (VEGF) secretion [26]. IL-8 which derived from macrophages, platelets, and epithelial cells could promote the angiogenesis of PC. Serum levels of IL-6, IL-8, IL-10, and IL-1RA were significantly increased in pancreatic cancer patients and were associated with worse survival rates, poor performance status. A panel of IP-10, IL-6, PDGF plus CA19-9 could discriminate PDAC patients from patients with pancreatic benign disease [27]. TNF-α is associated with acute and chronic inflammation and inflammation related to cancers [28]. In addition, increased expression of tumor-related inflammatory mediators and cytokines, such as TNF-α, IL-1, and IL-6 may stimulate the bone marrow to release neutrophils, resulting in an increase in the circulating neutrophil count and decrease in the circulating lymphocytopenia [29]. Neutrophils could promote growth and metastasis of tumors through secreting a variety of cytokines, including matrix metalloproteinase-9, chemokines and vascular endothelial growth factor (VEGF). It was reported that neutrophils could promote adhesion between circulating tumor cells and distant target organs through acting as an adhesive adapter, finally increasing the chance of distant metastasis. Moreover, neutrophil could also inhibit the antitumor immune function of natural killer cells and cytotoxic T cells [12]. Presently, it is believed that lymphocytes in the peripheral blood can cause synergistic cytotoxicity and play an anti-cancer role. Several subtypes of tumor infiltrating lymphocyte such as CD8 + T cells and memory T cells were associated with better outcomes of a variety of tumors, while regulatory T cells and Th17 cells were associated with progression and unfavorable prognosis of tumors [30]. Although different subset of T cells was associated with adverse prognosis of tumors, high level of absolute lymphocyte count was demonstrated to be associated with favorable prognosis of gastric cancer patients [31]. A study by Dominic et al. showed that inflammatory monocytes were lower in the bone marrow and higher in the blood of patients with resectable PC, and an increased blood-to-bone marrow monocyte ratio was a novel poor prognostic factor for PC [32]. Platelets are also involved in tumor development [33].

Meanwhile, patient’s nutritional status is associated with metabolic changes and immune status impairment. Circulating albumin and prealbumin are markers for evaluating nutritional status and immune status. Albumin can inhibit tumor progression by stabilizing DNA replication and enhancing the immune response [34]. The inflammatory factors may influence nutritional status through inhibition of appetite, alteration of gastrointestinal function, alteration of the carbohydrate metabolism and insulin resistance. Serum levels of IL-6 and IL-8 were inversely correlated to serum albumin and prealbumin. Serum IL-6 and IL-8 were highly expressed in patients with nutritional risk [35]. Genetic and pharmacological studies have revealed the key role of fibrinogen in determining the degree of local or systemic inflammation [36]. Fibrinogen is an important coagulation factor that can be recognized by a variety of integrin and non-integrin receptors on tumor, stromal, and inflammatory cells. These fibrinogen-mediated receptors are thought to control cell proliferation, apoptosis, cell migration, and the expression of inflammatory mediators [37].

In cancer, cytokines mediate signalling between cancer cells, and the cells of the TME, including PSCs, CAFs, endothelial cells, and a range of immune cells including macrophages, mast cells, neutrophils, and regulatory T-cells [38]. For example, glioblastoma (GBM) cells reduced lymphocyte infiltration by secreting immunosuppressive cytokines such as IL-10, IL-2, and TGF-β, and recruited and induced macrophages to become M2 phenotypes by secreting IL-10, IL-4, IL-6, macrophage–colony stimulating factor (M-CSF), TGF-β, and prostaglandin E2 (PGE2) [39]. Higher serum IL-8 and IL-6 levels were positively correlated with high NLR, modified glasgow prognostic score (mGPS), CRP-albumin ratio (CAR), and PLR [40, 41]. Fibrinogen induced the production of IL-6, IL-8, monocyte chemoattractant protein-1, vascular endothelial growth factor, angiopoietin-1 and type I collagen in pancreatic stellate cells [42]. CAR, NLR, and PNI were positively associated with IL-10, IL-23, and IL-1β [43]. Park et al. found moderate-to-strong correlations within circulating cytokines (TNF-α, IL-1β, IL-6, IL-8, IL-9, IL-10, and VEGF) as well as within systemic inflammatory markers (mGPS, NLR, and PNI) [44]. Higher mGPS was involved in increased plasma levels of IL-4, IL-6, IL-8 [45]. Patients with a low PNI exhibited high levels of TNF-αin advanced pancreatic cancer [46]. To sum up, there was a close relationship between systemic inflammatory markers and plasma cytokines.

Currently, routine measurement of serum inflammatory cytokines is not common in daily clinical practice. Many studies used inflammatory cell in the peripheral blood to reflect the systemic immune conditions of patients. In this study, we included HCs and patients with chronic pancreatitis, pancreatic serous/mucinous cystadenoma, solid pseudo-papilloma, and pancreatic neuroendocrine tumors. The results showed that serum albumin, prealbumin, and lymphocytes were significantly decreased, while fibrinogen, neutrophils, and monocytes were significantly increased in early-stage PC compared with HC and OPT. Our results provide supporting evidence that inflammation is emerging as a hallmark of early—stage cancer. Since neutrophil, monocyte, and lymphocyte counts are influenced by many factors, researchers are more inclined to use the ratio values between the two inflammation markers to explore the relationship between the ratio values and malignant tumor prognosis.

To date, many studies have shown that FAR, FPR, NLR, PLR, MLR, and PNI are predictive of outcomes in various types of cancer. For example, Michael et al. [47] found that an increased lymphocyte-to-monocyte ratio (LMR) was an independent prognostic factor for better cancer-specific survival in patients with PC (HR 0.70; *P* < 0.001). Qi et al. [36] showed that NLR, PLR, and LMR were independent predictors of survival in patients with advanced PC. Yi et al. [46] showed that a low PNI was associated with a systemic inflammatory response and was an independent poor prognostic factor for advanced PC. Fang et al. [16] reported that a high FAR was associated with poor OS in patients with locally advanced or metastatic PC. Xie et al. [48] found that high FPR was an independent poor prognostic factor for patients with stage I-III colorectal cancer (CRC). In addition, inflammatory indicators have important implications in cancer diagnosis. The combination of NLR, PLR, and CEA had a high diagnostic efficacy (AUC = 0.831, 95% CI = 0.807–0.852) for early-stage CRC. Zheng et al. [39] found that NLR + LMR and the derived neutrophil-to-lymphocyte ratio (dNLR) + LMR had good diagnostic performance in patients with glioma (AUC = 0.777 and 0.778, respectively). Wu et al. [49] showed that a combination of PLR and CEA had a better AUC of 0.780 than CEA alone for diagnosing gastric cancer. Lu et al. [50] found that the combination of CA199 and AFR distinguished PC from HC with an ROC of 0.932. Liu et al. [51] showed that combined circulating dNLR and Alb was an effective diagnostic biomarker for early stage PC (AUC = 0.931), and that dNLR could distinguish early-stage PC from HC (AUC 0.895) and from additional cancers (AUC 0.794). Similar to the above results, this study found that FAR, FPR, NLR, PLR, MLR, and FLR were higher in early-stage PC than in HC and OPT, whereas PNI was lower in patients with early-stage PC. These results indicate that inflammatory indicators could act as early diagnostic markers for PC. Moreover, ROC analysis indicated that the FAR, FPR, PLR, MLR, and PNI were promising diagnostic indicators. Among these inflammation markers, a combination of FAR, FPR, FLR, and CA199 could be used to differentiate early-stage PC from HC and OPT with a better AUC (0.964 and 0.924 in training sets). The results obtained in the training set were confirmed for two independent testing sets.

The inflammation indicators were similar over differences in sex, age, tumor size, differentiation, lymph nodes, and TNM stage, but varied greatly for different tumor locations. PHC always obstructs bile ducts, which leads to the levels of albumin (38.2 g/L vs 39.6 g/L, *P* = 0.007) and prealbumin (207.7 g/L vs 240.1 g/L, *P* = 0.0003) that are lower than PBTC. We further explored the discriminating ability of inflammation indicators for different tumor locations in early-stage PC. The combination of CA199, FAR, FPR, and FLR could better distinguish PHC from OPHT (AUC = 0.915) than PBTC from OPBTT (AUC = 0.894). Hence, for patients with pancreatic head tumors at the first medical visit, a combination of FAR, FPR, FLR, and CA199 would significantly guide the initial clinical diagnosis and aid in a more accurate final diagnosis.

Our study had some limitations. First, it was a retrospective analysis of data from a clinical trial and lacked prospective data. Second, although all patients were from two single-centers, we enrolled only a small number of patients from one center. Third, the participants in our study had no measurements of serum inflammatory cytokines such as IL-2, IL-6 and so on, we have no way to compared correlations between cytokine levels and inflammation markers ratios. However, despite several limitations, this study confirmed that FAR, FPR, FLR, and CA199 have a potential as diagnostic markers for early-stage PC. These results need to be confirmed in a multicenter, large-scale, prospective study.

## Conclusion

This study established that circulating inflammation markers ratios, especially FAR, FPR, and FLR, could be used as cost-effective diagnostic biomarkers for early-stage PC that improve the diagnostic accuracy over CA199. The combination of FAR, FPR, FLR, and CA199 was a potentially effective biomarker for distinguishing early -stage PC patients from HC and in differentiating early -stage PC patients from patients with OPT. The combination of FAR, FPR, FLR, and CA199 may be useful as a differential diagnostic marker for patients with pancreatic head cancer.

## Supplementary Information

**Additional file 1:** **Supplementary Figure 1.** The circulating inflammation markers in PC, HC, BPT, SPT, and PNET in testing set 1.

**Additional file 2:** **Supplementary Figure 2.** The inflammation markers ratios in PC, HC, BPT, SPT, and PNET in testing set 1.

**Additional file 3:** **Supplementary Figure 3.** Diagnostic value of single and combined inflammation markers ratios in early-stage PC.

**Additional file 4:** **Supplementary Figure 4.** Comparison of inflammation markers ratios in different clinical characteristics early-stage PC.

**Additional file 5:** **Supplementary Table 1.** ROC curve results based on FAR, FPR, MLR, PNI, FLR and CA199 for distinguishing PC patients from HC in testing set 1.

**Additional file 6:** **Supplementary Table 2.** ROC curve results based on FAR, FPR, FLR, and CA199 for distinguish PC patients from OPT in Testing set 1.

**Additional file 7:** **Supplementary Table 3.** ROC curve results based onFAR, FPR, NLR, PLR, LMR, PNI, FLR and CA199 for distinguishing PC from OPT in training sets 1.

## Data Availability

The datasets used and/or analysed during the current study available from the corresponding author on reasonable request.
