# Supplementary material for: Diagnostic value of combining preoperative inflammatory markers ratios with CA199 for patients with early-stage pancreatic cancer
Source: BMC Cancer. 2023 Mar 10;23:227. doi: 10.1186/s12885-023-10653-4 (PMC9999638; doi:10.1186/s12885-023-10653-4)
Supplement: Supplementary file 1 — Additional file 1: Supplementary Figure 1. The circulating inflammation markers in PC, HC, BPT, SPT, and PNET in testing set 1. [file 12885_2023_10653_MOESM1_ESM.pdf]

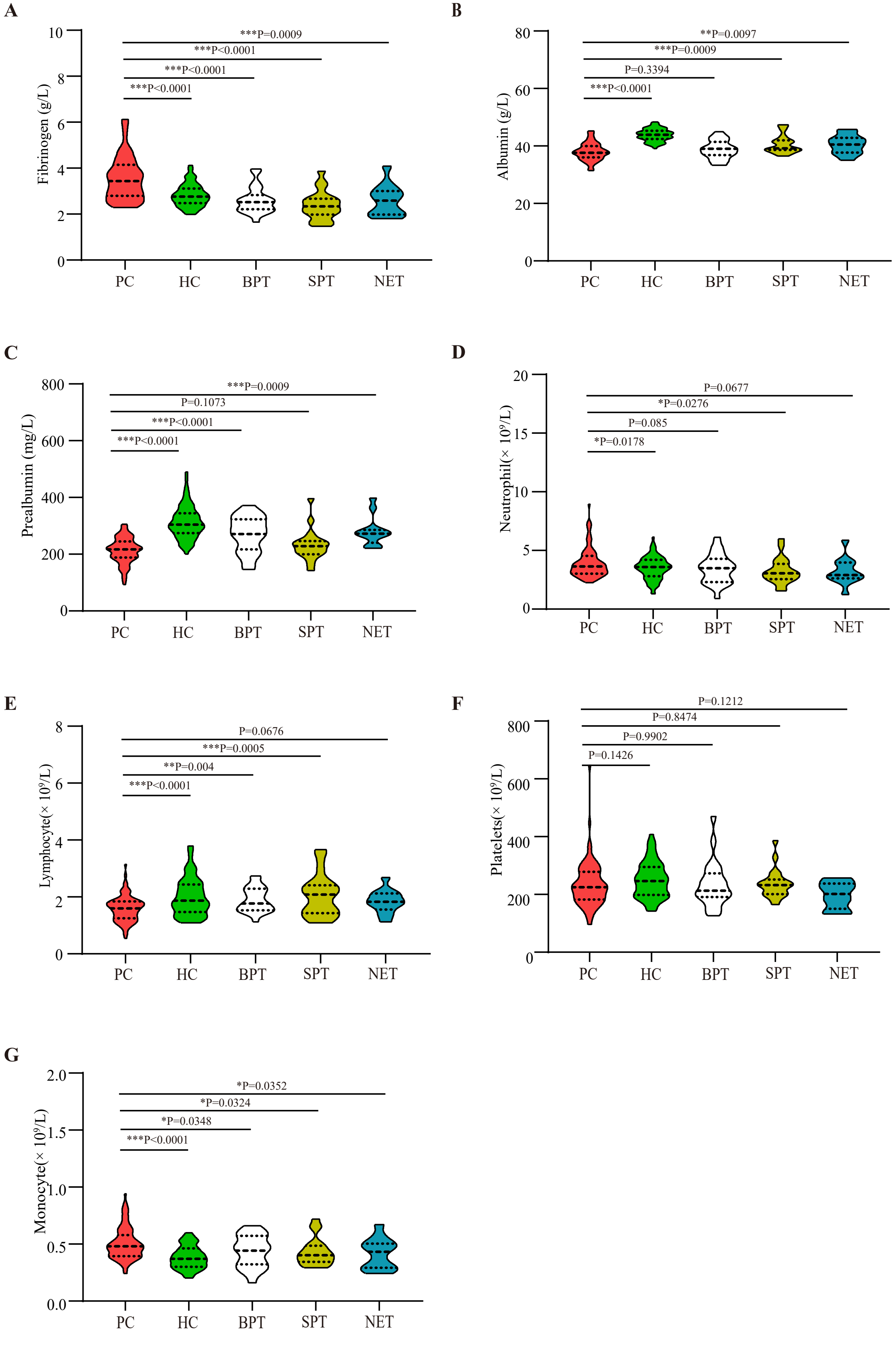
A
Fibrinogen (g/L)
***P=0.0009
***P<0.0001
***P<0.0001
***P<0.0001
PC
HC
BPT
SPT
NET
B
Albumin (g/L)
**P=0.0097
***P=0.0009
P=0.3394
***P<0.0001
C
Prealbumin (mg/L)
***P=0.0009
P=0.1073
***P<0.0001
***P<0.0001
D
Neutrophil(× 10^9/L)
P=0.0677
*P=0.0276
P=0.085
*P=0.0178
E
Lymphocyte(× 10^9/L)
P=0.0676
***P=0.0005
**P=0.004
***P<0.0001
F
Platelets(× 10^9/L)
P=0.1212
P=0.8474
P=0.9902
P=0.1426
G
Monocyte(× 10^9/L)
*P=0.0352
*P=0.0324
*P=0.0348
***P<0.0001

Supplementary Figure 1 The circulating inflammation markers in PC, HC, BPT, SPT, and PNET in testing set 1. The plasma fibrinogens(**A**), serum albumins (**B**), prealbumin (**C**), neutrophils (**D**), lymphocytes (**E**), platelets (**F**), and monocytes (**G**) in PC, HC, BPT, SPT, and PNET. Abbreviations: PC, pancreatic cancer; BPT, benign pancreas tumors; SPT, solid pseudo papilloma of the pancreas; PNET, patients with pancreatic neuroendocrine tumors; HC, healthy controls.
