# Supplementary material for: Diagnostic value of combining preoperative inflammatory markers ratios with CA199 for patients with early-stage pancreatic cancer
Source: BMC Cancer. 2023 Mar 10;23:227. doi: 10.1186/s12885-023-10653-4 (PMC9999638; doi:10.1186/s12885-023-10653-4)
Supplement: Supplementary file 2 — Additional file 2: Supplementary Figure 2. The inflammation markers ratios in PC, HC, BPT, SPT, and PNET in testing set 1. [file 12885_2023_10653_MOESM2_ESM.pdf]

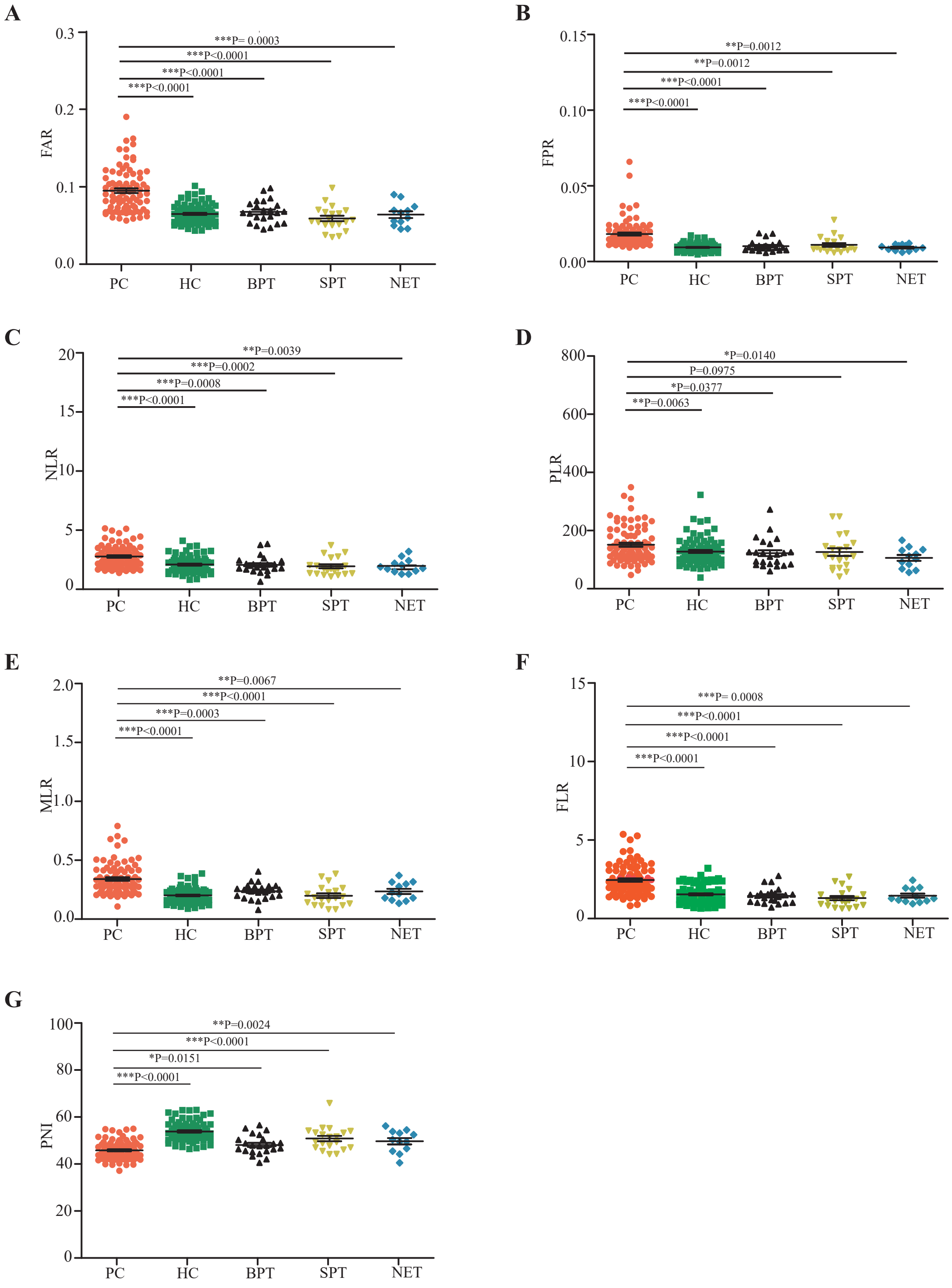

A
FAR
0.0
0.1
0.2
0.3
***P= 0.0003
***P<0.0001
***P<0.0001
***P<0.0001
PC
HC
BPT
SPT
NET
B
FPR
0.00
0.05
0.10
0.15
**P=0.0012
**P=0.0012
***P<0.0001
***P<0.0001
PC
HC
BPT
SPT
NET
C
NLR
0
5
10
15
20
**P=0.0039
***P=0.0002
***P=0.0008
***P<0.0001
PC
HC
BPT
SPT
NET
D
PLR
0
200
400
600
800
*P=0.0140
P=0.0975
*P=0.0377
**P=0.0063
PC
HC
BPT
SPT
NET
E
MLR
0.0
0.5
1.0
1.5
2.0
**P=0.0067
***P<0.0001
***P=0.0003
***P<0.0001
PC
HC
BPT
SPT
NET
F
FLR
0
5
10
15
***P= 0.0008
***P<0.0001
***P<0.0001
***P<0.0001
PC
HC
BPT
SPT
NET
G
PNI
0
20
40
60
80
100
**P=0.0024
***P<0.0001
*P=0.0151
***P<0.0001
PC
HC
BPT
SPT
NET

Supplementary Figure 2 The inflammation markers radios in PC, HC, BPT, SPT, and PNET in testing set 1. The FAR (**A**), FPR (**B**), NLR (**C**), PLR (**D**), MLR (**E**), FLR (**F**), and PNI (**G**) in PC, HC, BPT, SPT, and PNET. Abbreviations: PC, pancreatic cancer; BPT, benign pancreas tumors; SPT, solid pseudo papilloma of the pancreas; PNET, patients with pancreatic neuroendocrine tumors; HC, healthy controls; FAR, fibrinogen-to-albumin ratio; FPR, fibrinogen-to-prealbumin ratio; NLR, neutrophil-to-lymphocyte ratio; PLR, platelets-to-lymphocyte ratio; MLR monocytes-to-lymphocyte ratio; PNI, albumin +5×the lymphocyte count, FLR, fibrinogen-to- lymphocyte ratio.
