# Supplementary material for: Diagnostic value of combining preoperative inflammatory markers ratios with CA199 for patients with early-stage pancreatic cancer
Source: BMC Cancer. 2023 Mar 10;23:227. doi: 10.1186/s12885-023-10653-4 (PMC9999638; doi:10.1186/s12885-023-10653-4)
Supplement: Supplementary file 3 — Additional file 3: Supplementary Figure 3. Diagnostic value of single and combined inflammation markers ratios in early-stage PC. [file 12885_2023_10653_MOESM3_ESM.pdf]

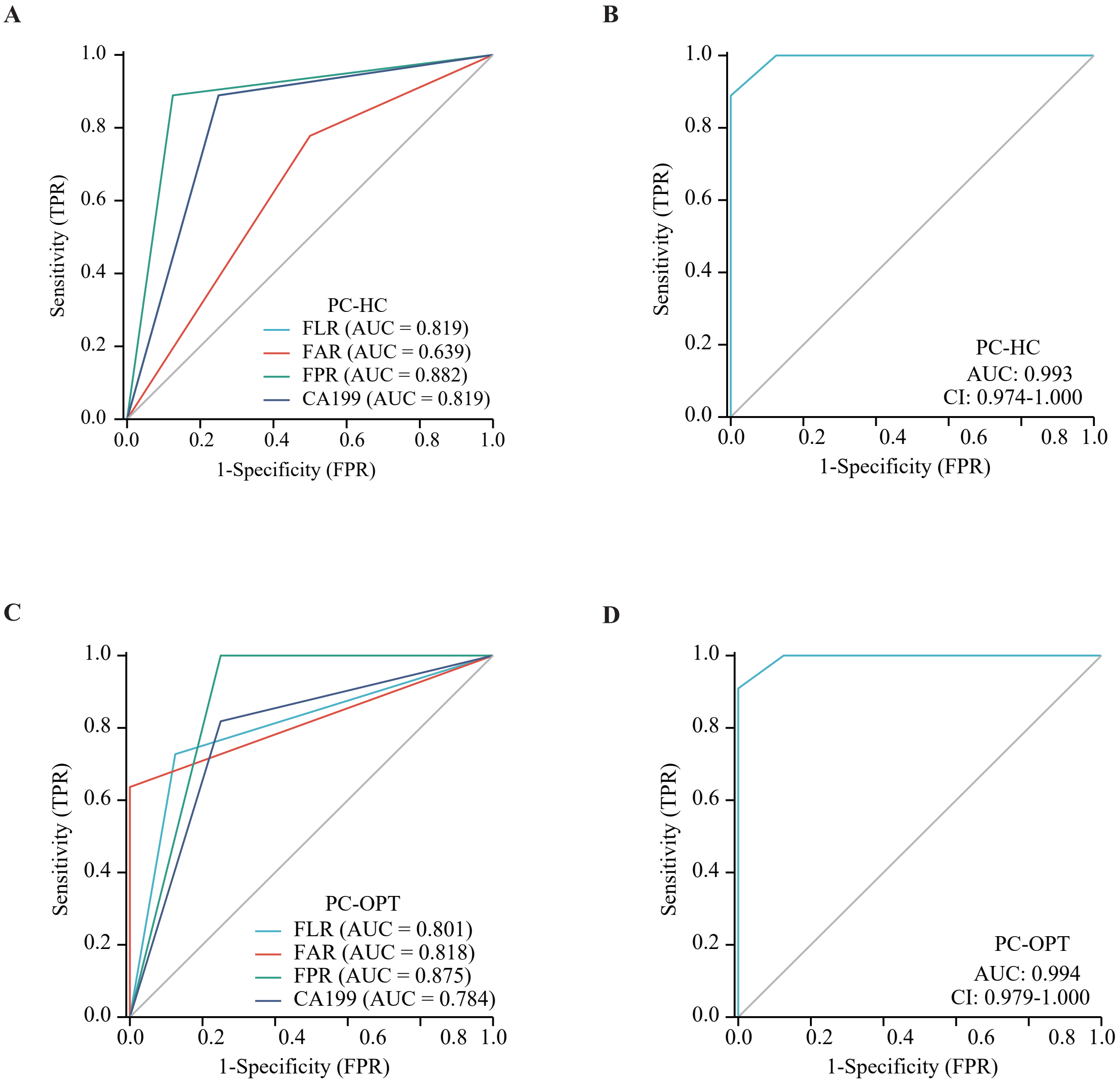

A
1.0
0.8
0.6
0.4
0.2
0.0
Sensitivity (TPR)
0.0
0.2
0.4
0.6
0.8
1.0
1-Specificity (FPR)
PC-HC
FLR (AUC = 0.819)
FAR (AUC = 0.639)
FPR (AUC = 0.882)
CA199 (AUC = 0.819)
B
Sensitivity (TPR)
1-Specificity (FPR)
PC-HC
AUC: 0.993
CI: 0.974-1.000
C
Sensitivity (TPR)
1-Specificity (FPR)
PC-OPT
FLR (AUC = 0.801)
FAR (AUC = 0.818)
FPR (AUC = 0.875)
CA199 (AUC = 0.784)
D
Sensitivity (TPR)
1-Specificity (FPR)
PC-OPT
AUC: 0.994
CI: 0.979-1.000

Supplementary Figure 3 Diagnostic value of single and combined inflammation markers radios in early-stage PC. **A** The ROC curve analysis of FAR, FPR, FLR, and CA199 between PC and HC in testing set 2. **B** The ROC curve analysis of combined FAR, FPR, FLR, and CA199 in PC and HC in testing set 2. **C** The ROC curve analysis of combined FAR, FPR, FLR, and CA199 between PC and OPT in testing set 2. **D** The ROC curve analysis of combined FAR, FPR, FLR, and CA199 between PC and OPT in testing set 2. Abbreviations: PC, pancreatic cancer; OPT, other pancreas tumors; HC, healthy controls; FAR, fibrinogen-to-albumin ratio; FPR, fibrinogen-to-prealbumin ratio; FLR, fibrinogen-to- lymphocyte ratio; ROC, receiver operating characteristic.
