# Supplementary material for: Diagnostic value of combining preoperative inflammatory markers ratios with CA199 for patients with early-stage pancreatic cancer
Source: BMC Cancer. 2023 Mar 10;23:227. doi: 10.1186/s12885-023-10653-4 (PMC9999638; doi:10.1186/s12885-023-10653-4)
Supplement: Supplementary file 4 — Additional file 4: Supplementary Figure 4. Comparison of inflammation markers ratios in different clinical characteristics early-stage PC. [file 12885_2023_10653_MOESM4_ESM.pdf]

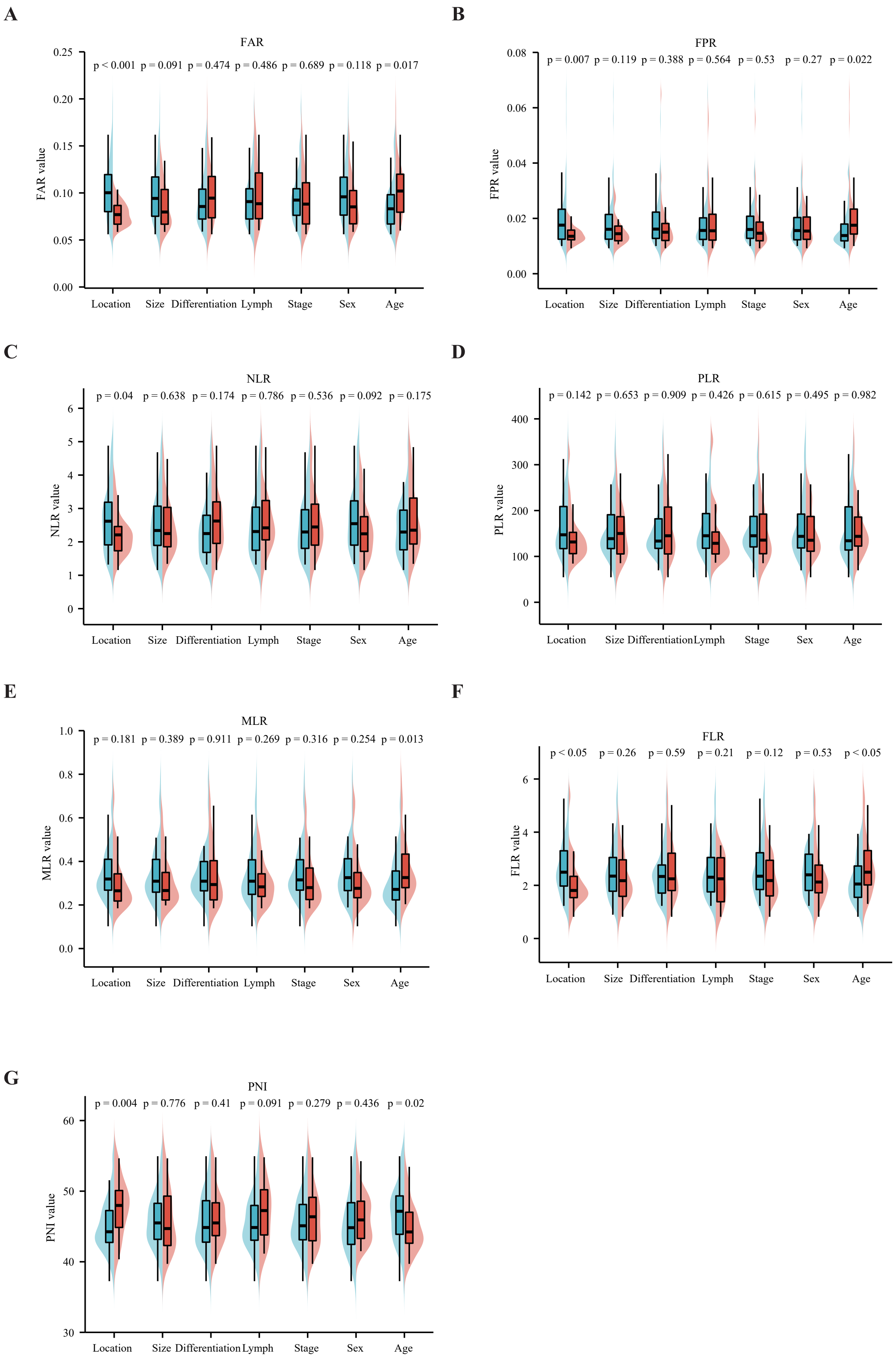
A
FAR
p < 0.001 p = 0.091 p = 0.474 p = 0.486 p = 0.689 p = 0.118 p = 0.017
FAR value
0.25
0.20
0.15
0.10
0.05
0.00
Location Size Differentiation Lymph Stage Sex Age
B
FPR
p = 0.007 p = 0.119 p = 0.388 p = 0.564 p = 0.53 p = 0.27 p = 0.022
FPR value
0.08
0.06
0.04
0.02
0.00
Location Size Differentiation Lymph Stage Sex Age
C
NLR
p = 0.04 p = 0.638 p = 0.174 p = 0.786 p = 0.536 p = 0.092 p = 0.175
NLR value
6
5
4
3
2
1
0
Location Size Differentiation Lymph Stage Sex Age
D
PLR
p = 0.142 p = 0.653 p = 0.909 p = 0.426 p = 0.615 p = 0.495 p = 0.982
PLR value
400
300
200
100
0
Location Size Differentiation Lymph Stage Sex Age
E
MLR
p = 0.181 p = 0.389 p = 0.911 p = 0.269 p = 0.316 p = 0.254 p = 0.013
MLR value
1.0
0.8
0.6
0.4
0.2
0.0
Location Size Differentiation Lymph Stage Sex Age
F
FLR
p < 0.05 p = 0.26 p = 0.59 p = 0.21 p = 0.12 p = 0.53 p < 0.05
FLR value
6
4
2
0
Location Size Differentiation Lymph Stage Sex Age
G
PNI
p = 0.004 p = 0.776 p = 0.41 p = 0.091 p = 0.279 p = 0.436 p = 0.02
PNI value
60
50
40
30
Location Size Differentiation Lymph Stage Sex Age

Supplementary Figure 4 Comparison of inflammation markers radios in different clinical characteristics early-stage PC. The FAR (**A**), FPR (**B**), NLR (**C**), PLR (**D**), MLR (**E**), FLR (**F**), and PNI (**G**) for different tumor locations, tumor sizes, differentiation, lymph nodes, stage, sex, and age in testing set 1. Abbreviations: PC, pancreatic cancer; FAR, fibrinogen-to-albumin ratio; FPR, fibrinogen-to-prealbumin ratio; NLR, neutrophil-to-lymphocyte ratio; PLR, platelets-to-lymphocyte ratio; MLR monocytes-to-lymphocyte ratio; PNI, albumin+5×the lymphocyte count; FLR, fibrinogen-to- lymphocyte ratio; Blue column: location, pancreatic head/tumor size≤4cm/ differentiation well / lymph node metastasis no /stage I/sex male/age ≤60; Red column: location, pancreatic body and tail/tumor size> 4cm/ differentiation poor / lymph node metastasis yes /stage II/sex male/age >60.
