# Supplementary material for: Diagnostic value of combining preoperative inflammatory markers ratios with CA199 for patients with early-stage pancreatic cancer
Source: BMC Cancer. 2023 Mar 10;23:227. doi: 10.1186/s12885-023-10653-4 (PMC9999638; doi:10.1186/s12885-023-10653-4)
Supplement: Supplementary file 5 — Additional file 5: Supplementary Table 1. ROC curve results based on FAR, FPR, MLR, PNI, FLR and CA199 for distinguishing PC patients from HC in testing set 1. [file 12885_2023_10653_MOESM5_ESM.docx]

| Marker | AUC (95%CI) | P - value | Cut-off | Sensitivity | Specificity |
| --- | --- | --- | --- | --- | --- |
| FAR | 0.770(0.708-0.833) | <0.0001 | 1.5 | 0.886 | 0.655 |
| FPR | 0.822(0.764-0.881) | <0.0001 | 1.5 | 0.835 | 0.810 |
| MLR | 0.787(0.725-0.849) | <0.0001 | 1.5 | 0.848 | 0.726 |
| PNI | 0.855(0.801-0.908) | <0.0001 | 1.5 | 0.911 | 0.798 |
| FLR | 0.773(0.708-0.837) | <0.0001 | 1.5 | 0.759 | 0.786 |
| CA199 | 0.860(0.808-0.913) | <0.0001 | 1.5 | 0.911 | 0.810 |
| CA199+FAR | 0.921(0.877-0.964) | <0.0001 | 1.043 | 0.835 | 0.940 |
| CA199+FPR | 0.934(0.894-0.973) | <0.0001 | 1.307 | 0.797 | 0.976 |
| CA199+MLR | 0.928(0.889-0.967) | <0.0001 | -0.590 | 0.911 | 0.810 |
| CA199+PNI | 0.952(0.921-0.983) | <0.0001 | 1.264 | 0.835 | 0.976 |
| CA199+FAR+FPR | 0.941(0.903-0.979) | <0.0001 | 0.097 | 0.861 | 0.929 |
| CA199+FAR+FPR+FLR | 0.947(0.913-0.981) | <0.0001 | -0.112 | 0.873 | 0.917 |
| CA199+FAR+ FPR+MLR+PNI | 0.975(0.957-0.993) | <0.0001 | 0.683 | 0.861 | 0.976 |

Supplementary Table 1 ROC curve results based on FAR, FPR, MLR, PNI, FLR and CA199 for distinguishing PC patients from HC in testing set 1.

Abbreviations: PC, pancreatic cancer; HC, heathy controls; ROC, receiver operating characteristic; AUC, area under the receiver operating characteristic curve; CI, confidence interval. FPR, fibrinogen-to-prealbumin ratio; FAR, fibrinogen-to-albumin ratio; NLR, neutrophil-to-lymphocyte ratio; PLR, platelets-to-lymphocyte ratio; MLR monocytes-to-lymphocyte ratio; PNI, albumin +5×the lymphocyte count; FLR, fibrinogen-to- lymphocyte ratio.

.
