# Supplementary material for: Diagnostic value of combining preoperative inflammatory markers ratios with CA199 for patients with early-stage pancreatic cancer
Source: BMC Cancer. 2023 Mar 10;23:227. doi: 10.1186/s12885-023-10653-4 (PMC9999638; doi:10.1186/s12885-023-10653-4)
Supplement: Supplementary file 6 — Additional file 6: Supplementary Table 2. ROC curve results based on FAR, FPR, FLR, and CA199 for distinguish PC patients from OPT in Testing set 1. [file 12885_2023_10653_MOESM6_ESM.docx]

Supplementary Table 2 ROC curve results based on FAR, FPR, FLR, and CA199 for distinguish PC patients from OPT in Testing set 1

| Marker | AUC (95%CI) | P - value | cut-off | Sensitivity | Specificity |
| --- | --- | --- | --- | --- | --- |
| FAR | 0.691(0.613-0.769) | <0.0001 | 1.5 | 0.727 | 0.655 |
| FPR | 0.823(0.758-0.888) | <0.0001 | 1.5 | 0.836 | 0.810 |
| FLR | 0.766(0.693-0.839) | <0.0001 | 1.5 | 0.745 | 0.786 |
| CA199 | 0.796(0.726-0.865) | <0.0001 | 1.5 | 0.782 | 0.810 |
| CA199+FAR | 0.861(0.800-0.922) | <0.0001 | -0.441 | 0.782 | 0.810 |
| CA199+FPR | 0.924(0.885-0.964) | <0.0001 | -0.432 | 0.964 | 0.690 |
| CA199+FAR+FPR | 0.925(0.884-0.966) | <0.0001 | -0.626 | 0.887 | 0.799 |
| CA199+FAR+FPR+FLR | 0.942(0.908-0.976) | <0.0001 | -0.068 | 0.836 | 0.869 |

Abbreviations: PC, pancreatic cancer; OPT, other pancreas tumors; ROC, receiver operating characteristic; AUC, area under the receiver operating characteristic curve; CI, confidence interval. FPR, fibrinogen-to-prealbumin ratio; FAR, fibrinogen-to-albumin ratio; NLR, neutrophil-to-lymphocyte ratio; PLR, platelets-to-lymphocyte ratio; MLR monocytes-to-lymphocyte ratio; PNI, albumin +5×the lymphocyte count; FLR, fibrinogen-to-lymphocyte ratio.
