# Supplementary material for: Diagnostic value of combining preoperative inflammatory markers ratios with CA199 for patients with early-stage pancreatic cancer
Source: BMC Cancer. 2023 Mar 10;23:227. doi: 10.1186/s12885-023-10653-4 (PMC9999638; doi:10.1186/s12885-023-10653-4)
Supplement: Supplementary file 7 — Additional file 7: Supplementary Table 3. ROC curve results based onFAR, FPR, NLR, PLR, LMR, PNI, FLR and CA199 for distinguishing PC from OPT in training sets 1. [file 12885_2023_10653_MOESM7_ESM.docx]

Supplementary Table 3 ROC curve results based on FAR, FPR, NLR, PLR, LMR, PNI, FLR and CA199 for distinguishing PC from OPT in training sets 1

| Marker | AUC (95%CI) | P - value | Cut-off | Sensitivity | Specificity |
| --- | --- | --- | --- | --- | --- |
| PBTC vs. OPBTT | | | | | |
| FAR | 0.706(0.638-0.775) | <0.0001 | 0.061 | 0.556 | 0.808 |
| FPR | 0.693(0.622-0.764) | <0.0001 | 0.011 | 0.675 | 0.641 |
| NLR | 0.585(0.508-0.662) | 0.0332 | 1.414 | 0.362 | 0.795 |
| PLR | 0.576(0.498-0.654) | 0.0574 | 105.930 | 0.613 | 0.577 |
| MLR | 0.529(0.452-0.606) | 0.469 | 0.273 | 0.731 | 0.359 |
| PNI | 0.601(0.522-0.679) | 0.0116 | 48.875 | 0.681 | 0.513 |
| FLR | 0.660(0.586-0.775) | <0.0001 | 1.216 | 0.469 | 0.795 |
| CA199 | 0.838(0.768-0.907) | <0.0001 | 34.46 | 0.931 | 0.744 |
| FAR+FPR+FLR | 0.714(0.646-0.783) | <0.0001 | 0.953 | 0.594 | 0.744 |
| CA199+FAR+FPR+FLR | 0.894(0.850-0.939) | <0.0001 | 0.613 | 0.956 | 0.692 |
| PHC vs. OPHT | | | | | |
| FAR | 0.750(0.685-0.815) | <0.0001 | 0.087 | 0.867 | 0.554 |
| FPR | 0.751(0.682-0.820) | <0.0001 | 0.014 | 0.750 | 0.669 |
| NLR | 0.767(0.701-0.833) | <0.0001 | 1.797 | 0.667 | 0.750 |
| PLR | 0.686(0.614-0.758) | <0.0001 | 123.969 | 0.600 | 0.700 |
| MLR | 0.766(0.704-0.828) | <0.0001 | 0.271 | 0.733 | 0.681 |
| PNI | 0.709(0.638-0.781) | <0.0001 | 47.175 | 0.750 | 0.650 |
| FLR | 0.824(0.768-0.879) | <0.0001 | 2.006 | 0.883 | 0.658 |
| CA199 | 0.855(0.809-0.901) | <0.0001 | 27.21 | 0.833 | 0.827 |
| FAR+FPR+FLR | 0.834(0.779-0.890) | <0.0001 | -1.528 | 0.833 | 0.715 |
| CA199+FAR+FPR+FLR | 0.915(0.874-0.956) | <0.0001 | -1.055 | 0.883 | 0.858 |

Abbreviations: PC, pancreatic cancer; OPT, other pancreatic tumor; PHC, pancreatic head cancer patients; OPHT, other pancreatic head tumor; PBTC, pancreatic body or tail cancer patients; OPBTT, other pancreatic body or tail tumor; ROC, receiver operating characteristic; AUC, area under the receiver operating characteristic curve; CI, confidence interval. FPR, fibrinogen-to-prealbumin ratio; FAR, fibrinogen-to-albumin ratio; FLR, fibrinogen-to-lymphocyte ratio.
